# Genetic Risk Score for Intracranial Aneurysms: Prediction of Subarachnoid Hemorrhage and Role in Clinical Heterogeneity

**DOI:** 10.1161/STROKEAHA.122.040715

**Published:** 2023-01-19

**Authors:** Mark K. Bakker, Jos P. Kanning, Gad Abraham, Amy E. Martinsen, Bendik S. Winsvold, John-Anker Zwart, Romain Bourcier, Tomonobu Sawada, Masaru Koido, Yoichiro Kamatani, Sandrine Morel, Philippe Amouyel, Stéphanie Debette, Philippe Bijlenga, Takiy Berrandou, Santhi K. Ganesh, Nabila Bouatia-Naji, Gregory Jones, Matthew Bown, Gabriel J.E. Rinkel, Jan H. Veldink, Ynte M. Ruigrok

**Affiliations:** Department of Neurology and Neurosurgery, University Medical Center Utrecht Brain Center, Utrecht University, the Netherlands (M.K.B., J.P.K., G.J.E.R., Y.M.R., J.H.V.).; Cambridge Baker Systems Genomics Initiative, Baker Heart and Diabetes Institute, Melbourne, VIC, Australia (G.A.).; Department of Clinical Pathology, University of Melbourne, Parkville, VIC, Australia (G.A.).; Department of Research and Innovation, Division of Clinical Neuroscience, Oslo University Hospital, Norway (A.E.M., B.S.W., J.-A.Z.).; Institute of Clinical Medicine, Faculty of Medicine, University of Oslo, Norway (A.E.M., J.-A.Z.).; K.G. Jebsen Center for Genetic Epidemiology, Department of Public Health and Nursing, NTNU, Norwegian University of Science and Technology, Trondheim, Norway (A.E.M., B.S.W., J.-A.Z.).; Department of Neurology, Oslo University Hospital, Norway (B.S.W.).; Université de Nantes, CHU Nantes, INSERM, CNRS, l’institut du thorax, France (R.B.).; CHU Nantes, Department of Neuroradiology, France (R.B.).; Graduate School of Frontier Sciences, The University of Tokyo, Japan (T.S., Y.K.).; Laboratory for Statistical and Translational Genetics, RIKEN Center for Integrative Medical Sciences, Yokohama, Japan (M.K.).; Department of Cancer Biology, Institute of Medical Science, The University of Tokyo, Japan (M.K.).; Neurosurgery Division, Department of Clinical Neurosciences, Faculty of Medicine, Geneva University Hospitals, Switzerland (P.B., S.M.).; Department of Pathology and Immunology, Faculty of Medicine, University of Geneva, Switzerland (S.M.).; LabEx DISTALZ-U1167, RID-AGE-Risk Factors and Molecular Determinants of Aging-Related Diseases, University of Lille, Lille, France; Inserm U1167, Lille, France; Centre Hospitalier Universitaire Lille, Lille, France; Institut Pasteur de Lille, Lille, France (P.A.).; University of Bordeaux, INSERM, Bordeaux Population Health Center, UMR1219, Bordeaux, France (S.D.).; Bordeaux University Hospital, Department of Neurology, Institute of Neurodegenerative Diseases, France (S.D.).; PARCC, INSERM, Université de Paris, France (T.B., N.B.-N.).; Division of Cardiovascular Medicine, Department of Internal Medicine (S.K.G.), University of Michigan Medical School, Ann Arbor.; Department of Human Genetics (S.K.G.), University of Michigan Medical School, Ann Arbor.; Department of Surgery, University of Otago, Dunedin, New Zealand (G.J.).; Department of Cardiovascular Sciences and National Institute for Health Research (M.B.); Leicester Biomedical Research Centre (M.B.); University of Leicester, Glenfield Hospital, United Kingdom (M.B.).

**Keywords:** aneurysmal subarachnoid hemorrhage, genetic heterogeneity, genetics, intracranial aneurysm, risk assessment

## Abstract

Recently, common genetic risk factors for intracranial aneurysm (IA) and aneurysmal subarachnoid hemorrhage (ASAH) were found to explain a large amount of disease heritability and therefore have potential to be used for genetic risk prediction. We constructed a genetic risk score to (1) predict ASAH incidence and IA presence (combined set of unruptured IA and ASAH) and (2) assess its association with patient characteristics.

**Methods:**

A genetic risk score incorporating genetic association data for IA and 17 traits related to IA (so-called metaGRS) was created using 1161 IA cases and 407 392 controls from the UK Biobank population study. The metaGRS was validated in combination with risk factors blood pressure, sex, and smoking in 828 IA cases and 68 568 controls from the Nordic HUNT population study. Furthermore, we assessed association between the metaGRS and patient characteristics in a cohort of 5560 IA patients.

**Results:**

Per SD increase of metaGRS, the hazard ratio for ASAH incidence was 1.34 (95% CI, 1.20–1.51) and the odds ratio for IA presence 1.09 (95% CI, 1.01–1.18). Upon including the metaGRS on top of clinical risk factors, the concordance index to predict ASAH hazard increased from 0.63 (95% CI, 0.59–0.67) to 0.65 (95% CI, 0.62–0.69), while prediction of IA presence did not improve. The metaGRS was statistically significantly associated with age at ASAH (β=−4.82×10^−3^ per year [95% CI, −6.49×10^−3^ to −3.14×10^−3^]; *P*=1.82×10^−8^), and location of IA at the internal carotid artery (odds ratio=0.92 [95% CI, 0.86–0.98]; *P*=0.0041).

**Conclusions:**

The metaGRS was predictive of ASAH incidence, although with limited added value over clinical risk factors. The metaGRS was not predictive of IA presence. Therefore, we do not recommend using this metaGRS in daily clinical care. Genetic risk does partly explain the clinical heterogeneity of IA warranting prioritization of clinical heterogeneity in future genetic prediction studies of IA and ASAH.

Rupture of an intracranial aneurysm (IA) leads to aneurysmal subarachnoid hemorrhage (ASAH), a severe type of stroke causing death in one-third of the cases, and permanent disability in another third.^1^ It is one of the few cardiovascular diseases in which women are at higher risk than men and is caused by a complex interplay of genetic factors and environmental risk factors,^2,3^ including smoking and hypertension.^4,5^ Aneurysmal rupture can be prevented by endovascular treatment or surgery, with relatively low risk of complications compared to the high case fatality and morbidity of ASAH.^6^ Therefore, prediction of ASAH has high potential in reducing disease burden.

**See related article, p 819**

Genetic risk scores (GRSs) showed potential in risk prediction of common diseases.^7^ New techniques improved prediction potential of GRSs by (1) providing methods to include a large number of genetic variants^8^ and (2) combining GRSs for multiple traits (a so-called metaGRS), leading to improved prediction of, among others, coronary artery disease and ischemic stroke.^9,10^ These advances, combined with the finding that single-nucleotide polymorphisms (SNPs) capture a substantial amount of heritability of IA (SNP-based heritability of 21.6%) in the latest genome-wide association study (GWAS) of IA,^2^ provide an opportunity for genetic risk prediction of IA.

A broad spectrum of clinical heterogeneity of IAs exists, including number, size, and different locations of IAs.^11,12^ A GRS constructed with only 7 SNPs was higher in patients with IAs at the middle cerebral artery compared to those with IAs at other locations, in a cohort of 1691 IA patients.^13^ In a cohort of 4890 patients of whom 109 had an unruptured IA (UIA), a 10-SNP GRS was associated with aneurysmal diameter and volume.^14^ These studies show a potential link between genetic predisposition and patient characteristics, but additional studies using larger populations and assessing SNPs across the genome are warranted.

We created a metaGRS for IA that incorporates GWAS summary statistics for IA together with summary statistics for other stroke subtypes and risk factors for IA, to assess its predictive performance for ASAH incidence and IA presence. In addition, we assessed how the metaGRS associates with clinical characteristics of IA patients.

## Methods

This article adheres to the PRS-RS reporting guidelines.^15^

### Ethics Statement

Written informed consent was obtained from all participants. The Biobanks Review Committee of the University Medical Center Utrecht gave ethical approval for the use of genotype and phenotype data of these participants.

### Data Availability

The metaGRS per-SNP weights are available here: https://doi.org/10.6084/m9.figshare.19672272 (including UK Biobank) and here: https://doi.org/10.6084/m9.figshare.19672269 (excluding UK Biobank).

### Methods Overview

**Figure 1.**
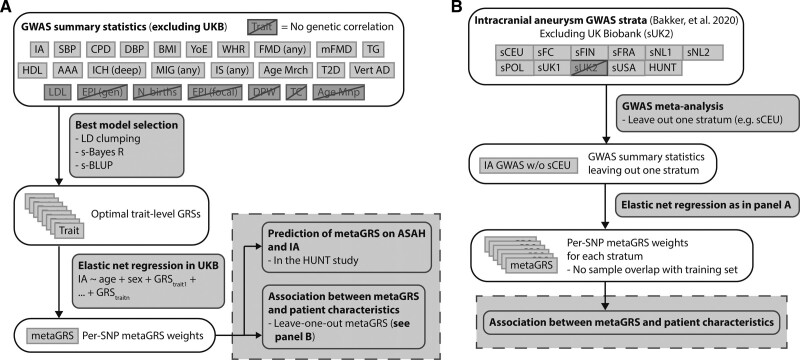
**Overview of constructing the metaGRS. A**, Steps to create the metaGRS used for prediction in the HUNT (Nordic HUNT study). **B**, Steps to create intracranial aneurysm (IA) GWAS summary statistics to be included in an adjusted metaGRS version for phenotype-genotype correlation analysis. Single IA GWAS strata were excluded from the IA GWAS in a leave-one-out manner prior to the “Best model selection” step in **A**. AAA indicates abdominal aortic aneurysm; Age Mnp, age at menopause; Age Mrch, age at menarche; BMI, body mass index; CPD, cigarettes per day; DBP, diastolic blood pressure; DPW, alcoholic drinks per week; EPI (focal), focal epilepsy; EPI (gen), generalized epilepsy; FMD (any), any fibromuscular dysplasia; HDL, high-density lipoprotein; HUNT, Nordic HUNT study; ICH (deep), deep intracerebral hemorrhage; IS (any), any ischemic stroke; LDL, low-density lipoprotein; mFMD, multifocal fibromuscular dysplasia; MIG (any), any migraine; N births, number of births; SBP, systolic blood pressure; sCEU, stratum of mixed European ancestry; sFC, French Canada; sFIN, Finnish; sFRA, France; sNL1; sNL2, Netherlands; sPOL, Poland; sUK1; sUK2, United Kingdom; sUSA, United States of America; T2D, type II diabetes; TC, total cholesterol; TG, triglycerides; Vert AD, vertebral artery dissection; WHR, waist-to-hip ratio; and YoE, years of education.

Figure 1 shows an overview of the study methods. In short, trait-level GRSs were constructed using summary statistics of the largest publicly available GWASs of IA and related traits (N=7495 cases and 71 934 controls of European ancestry; Table S1). Optimal GRS model selection, and combining these GRSs into a metaGRS, was performed using the UK Biobank, a prospective population-based cohort including 1161 IA patients (959 with ASAH and 202 with UIA) and 407 392 controls (Figure 1A; Table 1). Predictive performance of the metaGRS was assessed in the HUNT prospective population-based cohort study including 828 IA patients (318 with ASAH and 510 with UIA) and 68 568 controls (Table 1). Associations between metaGRS and patient characteristics were assessed in the well-phenotyped cohort of the international stroke genetics consortium (ISGC, www.strokegenetics.org) IA working group (ISGC-IA), including 5560 IA patients of whom 3918 with ASAH and 1642 with UIA (Table S2).^16^

**Table 1.**
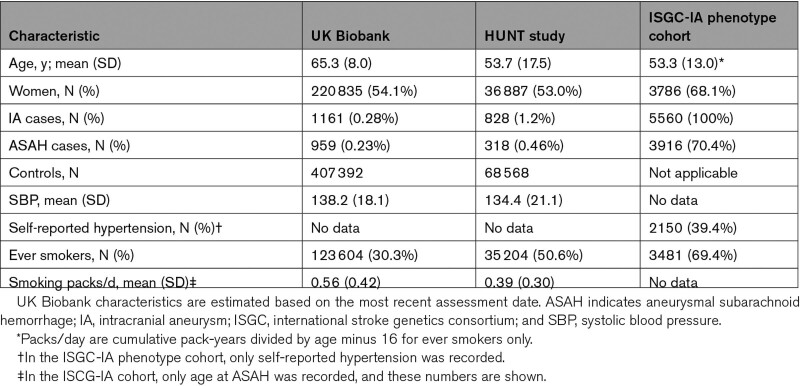
Baseline Characteristics of the Study Populations

### Constructing the metaGRS

Methods of the metaGRS construction are shown in Figure 1A. Summary statistics of large GWASs of IA and 24 traits with an established or putative association with IA were obtained. These 24 traits included (references and study info in Table S1): (1) established risk factors for IA and/or ASAH, being diastolic and systolic blood pressure (SBP), smoking (cigarettes per day), and alcohol consumption (drinks per week)^4,5,17–19^; (2) suggestive risk factors including those related to female hormones (age at menarche, age at menopause and number of births),^20^ and cardiovascular disease risk (diabetes type II, body mass index, waist-to-hip ratio, low- and high density lipoprotein levels, total cholesterol, and triglyceride levels),^19,21,22^ migraine,^23^ epilepsy (focal and generalized), years of education^24^; and (3) diseases genetically correlated with IA, being intracerebral hemorrhage, ischemic stroke, abdominal aortic aneurysms, and fibromuscular dysplasia (multifocal and any type); and (4) vascular disease vertebral artery dissections which showed a nominal genetic correlation with IA before.^2^ Only individuals of European-ancestry were included while UK Biobank participants were excluded. Data pre-processing steps are described in the Supplemental Material.

For traits genetically correlated with IA (*P*<0.05) trait-level GRSs were created (Supplemental Material) using 3 methods: LD-based clumping with 9 different LD thresholds, summary statistics-based best linear unbiased predictor^25^ and summary statistics-based BayesR^8^ (Figure 1A; Supplemental Material). To assess whether the trait-level GRSs captured risk of the respective trait, we tested whether the optimal trait-level GRS was associated with that trait in the UK Biobank, and tested whether including the trait-level GRS led to a higher area under the curve (AUC) or *R*^2^ compared to a reference model (for example, the trait-level GRS for SBP being associated with SBP in the UK biobank). Samples with a missing genotype value for a variant were ignored for association with that variant, while samples with missing phenotype were excluded (Supplemental Material for detailed methods).

For each trait, the trait-level GRS with the highest Nagelkerke pseudo-R^2^ in predicting IA status in the UK Biobank cohort was selected (Table S3) and subsequently jointly analyzed in an elastic-net regression to obtain per-trait weights (Figure 1A). Traits with an effect in the elastic-net regression were included in the metaGRS. Trait-level SNP weights were scaled according to the per-trait elastic net weights and population standard deviation, and then summed over traits to create metaGRS SNP weights (see Supplemental Material for extensive methods). These analyses were performed for the whole cohort and for men and women separately. A separate GRS was constructed only considering the IA GRS, to assess potential added value of incorporating genetic association data of multiple traits in a metaGRS compared to an IA-only GRS.

### Prediction of ASAH and IA by the metaGRS

Prediction by the metaGRS was evaluated in the HUNT study in 2 models: (1) for ASAH incidence using cox regression with age at ASAH as outcome and age at last assessment for controls as censoring time, and (2) for IA presence (including both UIA and ASAH) using a logistic regression with IA case-control status as outcome. For logistic regression; age, SBP, and average smoking packs per day since age 16 (SBP and smoking as 3-knot polynomial spline), were included as covariates. For Cox regression, age was left out as covariate (further details described in the Supplemental Material). The added value of specific predictors was assessed using various models: (1) a reference model (sex and age), clinical model (only sex, age, SBP, and smoking), (2) a reference+metaGRS model, (3) a full model (clinical model+metaGRS), and (4) models leaving out a single predictor from the full model. Predictive value was determined in the HUNT study using a metaGRS created with GWAS summary statistics for IA leaving out samples from the HUNT study (Figure 1B). The statistical significance of comparing the different models in their predictive ability was assessed using a DeLong test for IA presence (R package Daim), and by net reclassification index and integrated discrimination index for ASAH incidence (R package survIDINRI).

### Association Between metaGRS and Patient Characteristics

The ISGC-IA phenotype cohort of 5560 IA patients was used to determine the association of the metaGRS with the following patient characteristics: sex, smoking status (ever or never), self-reported hypertension, age at ASAH, and family history of IA (≥1 first degree relative with ASAH and/or UIA), IA location, number of IAs (single versus multiple), rupture status (UIA versus ASAH), and aneurysmal size at rupture. Locations of IA were grouped: (1) internal carotid artery (ICA) including the ICA, ophthalmic artery, and cavernous artery, (2) posterior communicating artery, (3) anterior cerebral arteries including the A1 anterior segment, anterior communicating artery, and A2 segment, (4) middle cerebral artery, and (5) posterior circulation (PC), including the vertebrobasilar system. IAs at other locations were excluded from these analyses.

Since most cases of the ISGC-IA phenotype cohort were included in the IA GWAS, we created stratum-specific metaGRSs leaving out samples from the ISGC-IA phenotype cohort one GWAS stratum at a time from the IA GWAS summary statistics, resulting in 9 metaGRS versions (Figure 1B; Supplemental Material). To control for differences in metaGRS versions between strata, we used the different cohorts of the ISGC-IA phenotype cohort (Table S4) as covariate in all subsequent analyses.

We calculated associations between metaGRS and patient characteristics, correcting for sex and cohort using a generalized linear model. We tested whether statistically significantly associated phenotypes were independently associated from one another using a multivariate model. For each phenotype, samples with missing values were excluded for analysis of that specific phenotype. In analyses studying the association with IA location, we included only patients with one IA. Statistical analyses were done in R 4.1.2. Statistical significance was determined by Bonferroni correction for the number of phenotypes in the primary analyses: rupture status, sex, family history, IA multiplicity, 5 locations, age at ASAH, and size at rupture (*P*<0.05/11). More details on the statistics are described in the Supplemental Material.

## Results

### Constructing the metaGRS

Seventeen out of 24 traits showed genetic correlation with IA (*P*<0.05; Figure 1; Table S5). All trait-level GRSs, except for the GRS of intracerebral hemorrhage, were associated with their respective phenotypes in the UK Biobank, validating the method for obtaining the trait-level GRSs (Table S6). Elastic-net regression weights for each trait-level GRS are shown in Table S7. In total, 7 078 955 SNPs were included in the metaGRS. In separate models trained in only men or women in the UK Biobank, 6 618 190 and 6 671 269 SNPs remained, respectively.

### Prediction of ASAH by the metaGRS

**Figure 2.**
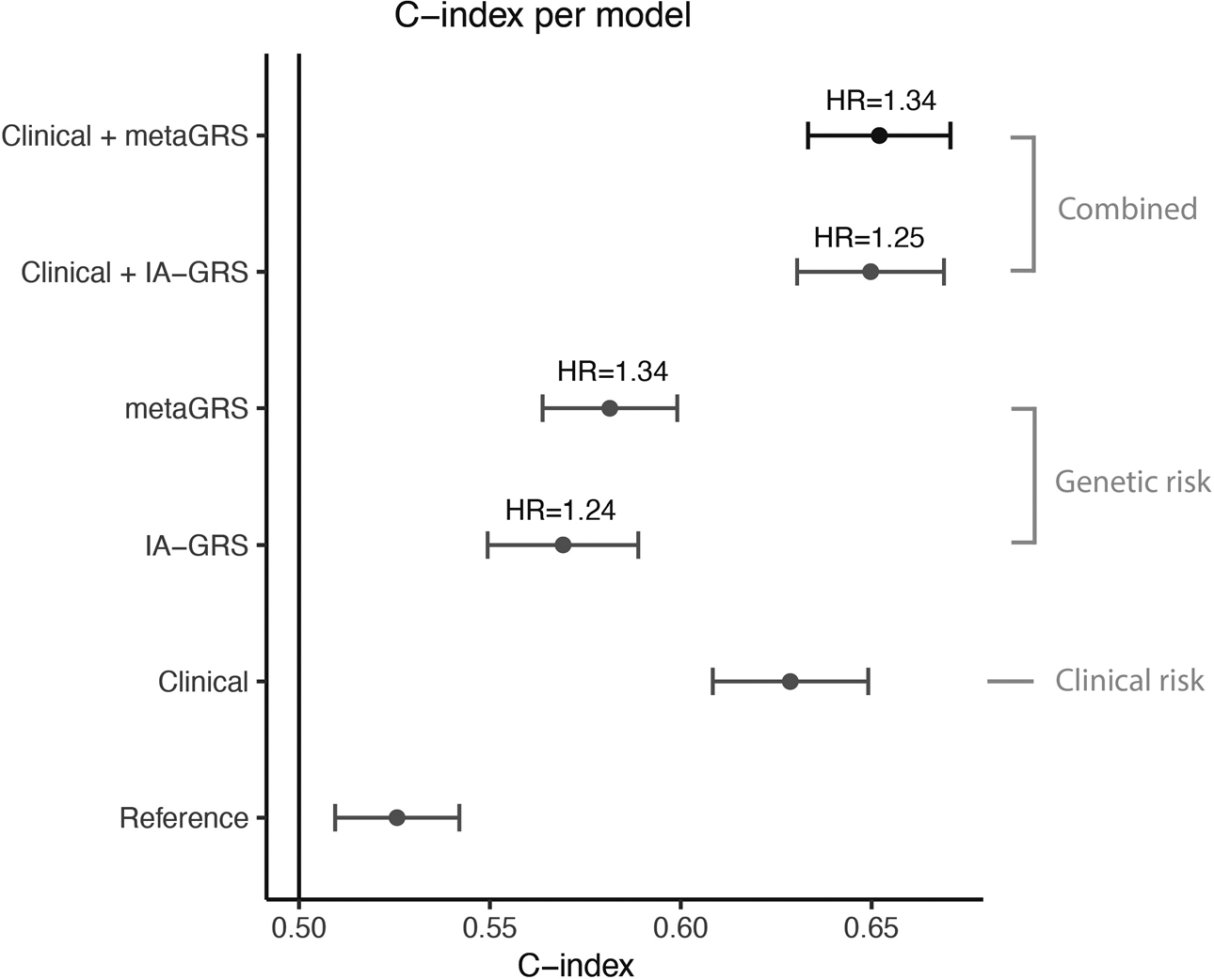
**Prediction of aneurysmal subarachnoid hemorrhage (ASAH) using the metaGRS in the HUNT study.** *C*-index according to different combinations of clinical risk factors and metaGRS are shown. Error bars denote 95% CIs. HR: hazard ratio per SD-specified genetic risk score (GRS). Reference: model including only sex. Clinical: model including sex, intracranial aneurysm (IA), systolic blood pressure, and smoking.

Characteristics of the HUNT study validation cohort are shown in Table 1. The metaGRS ranged from −0.83 to +0.50, with mean −0.22 and SD 0.14. The metaGRS showed improved prediction of ASAH incidence compared to a reference model including only sex (hazard ratio [HR]=1.34 [95% CI, 1.20–1.51]; *P*=6.1×10^−7^; Table S8). The C-index increased from 0.53 (95% CI, 0.49–0.56) to 0.58 (95% CI, 0.55–0.62) upon including the metaGRS to the reference model *P*_integrated_discrimination_index_<1×10^−8^; *P*_net_reclassification_index_=0.14; Table S9). The metaGRS seemed to outperform a GRS constructed using only summary statistics of IA (HR reference model+IA-only GRS=1.25 [95% CI, 1.12–1.41], C-index=0.57 [95% CI, 0.53–0.61]). Maximum prediction was reached upon including the metaGRS on top of clinical risk factors, where the C-index increased from 0.63 (95% CI, 0.59–0.67) to 0.65 ([95% CI, 0.62–0.69]; *P*_integrated_discrimination_index_=0.09; *P*_net_reclassification_index_=0.25; Table S9; Figure 2).

In the model trained in women in the UK Biobank and validated in women in the HUNT study, the metaGRS alone had a greater effect compared to the model of both women and men and the model of men only (women: HR per SD of metaGRS=1.36 [95% CI, 1.18–1.60], men: 1.12 [95% CI, 0.93–1.34]; Figure S1; Table S8). Similarly, clinical risk factors combined provided better prediction in women, and worse in men (women: *C*-index=0.71 [95% CI, 0.67–0.75], men: 0.57 [95% CI, 0.52–0.62]; Table S9). Furthermore, metaGRS outperformed IA-only GRS in women, similar to what was observed in the whole cohort (IA-only GRS in women: HR=1.30 [95% CI, 1.11–1.51]).

### Prediction of IA by the metaGRS

In prediction of IA presence (either UIA or ASAH) in the HUNT study, the metaGRS provided a small but statistically significant effect (odds ratio [OR]=1.09 [95% CI, 1.01–1.18]; Table S10). The metaGRS did not improve prediction above a model including clinical risk factors (AUC clinical model=0.76 [95% CI, 0.75–0.78], AUC clinical+metaGRS=0.76 [95% CI, 0.75–0.78]; *P*-value of difference=0.15; Figure S2; Table S11). Only the predictors age and SBP showed independent added value (ΔAUC excluding age versus full model=−0.067 [95% CI, −0.083 to −0.051]; *P*=1.1×10^−16^, ΔAUC excluding SBP=−0.01 [95% CI, −0.005 to −0.018]; *P*=2.9×10^−4^).

### Association Between metaGRS and Patient Characteristics

**Figure 3.**
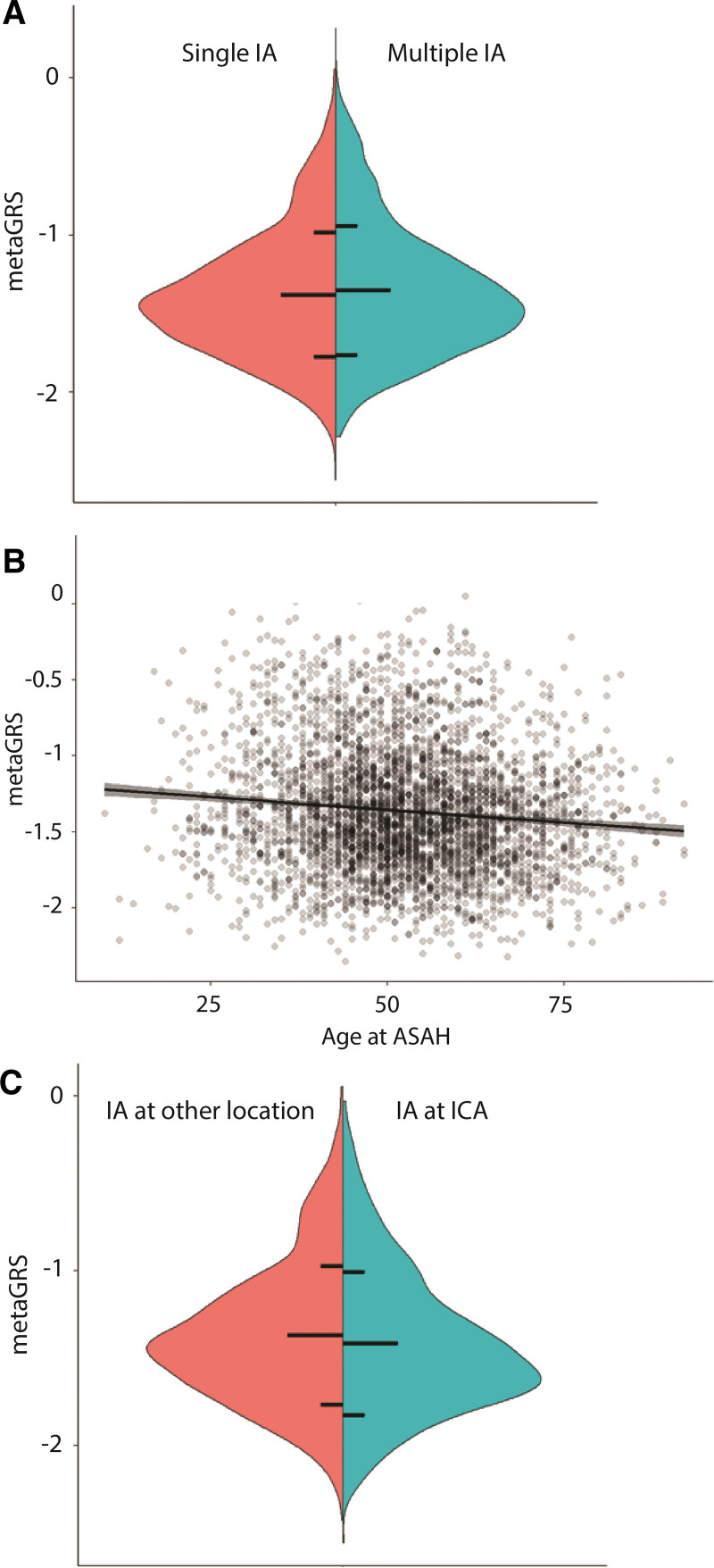
**Association of metaGRS with patient characteristics.** The metaGRS was transformed to mean 0, variance 1. **A**, metaGRS according to single or multiple intracranial aneurysms (IAs). Horizontal lines correspond to population mean (middle line), and mean±one population SD. **B**, Effect of age at aneurysmal subarachnoid hemorrhage (ASAH) on metaGRS. Line denotes regression line, with shared area being the 95% CI. **C**, Effect of having an IA at the internal carotid artery (ICA) versus other locations, on the metaGRS.

In the ISGC-IA phenotype cohort, patients with multiple IAs had a higher metaGRS than patients with a single IA, with nominal statistical significance (OR=1.05 [95% CI, 1.01–1.09]; *P*=0.010; Table 2; Figure 3A). Younger age at ASAH was associated with a higher metaGRS (β=−4.82×10^−3^ per year [95% CI, −6.49×10^−3^ to −3.14×10^−3^; *P*=1.82×10^−8^; Figure 3B). Accordingly, the effect of 1 SD increase of metaGRS on age at ASAH was −1.70 (95% CI, −2.30 to −1.11) years. Assuming a linear effect this equates to patients with a top 5% metaGRS suffering ASAH on average 2.80 (95% CI, 1.83–3.77) years earlier compared with patient with a mean metaGRS, while this is 3.96 (95% CI, 2.59–5.34) years earlier in patients with a top 1% versus mean metaGRS. Of all aneurysmal locations, only patients with an IA at the ICA had lower genetic risk (OR=0.92 [95% CI, 0.86 to 0.98]; *P*=0.0041; Figure 3C; Figures S3 through S7). This effect reduced and was not statistically significant anymore when considering ruptured IAs only (OR=0.94 [95% CI, 0.86–1.03]; *P*=0.16; Figures S8 through S12). No effect was observed for sex, positive family history, rupture status of an IA, or aneurysmal size at rupture (Table 2; Figures S13 through S16). A higher metaGRS was associated with hypertension (OR=1.10 [95% CI, 1.06–1.14]; *P*=3.82×10^−7^) and ever smokers (OR=1.14 [95% CI, 1.10–1.18]; *P*=9.30×10^−10^; Table S12; Figures S17 and S18), which is expected due to including summary statistics for these traits in the metaGRS.

**Table 2.**
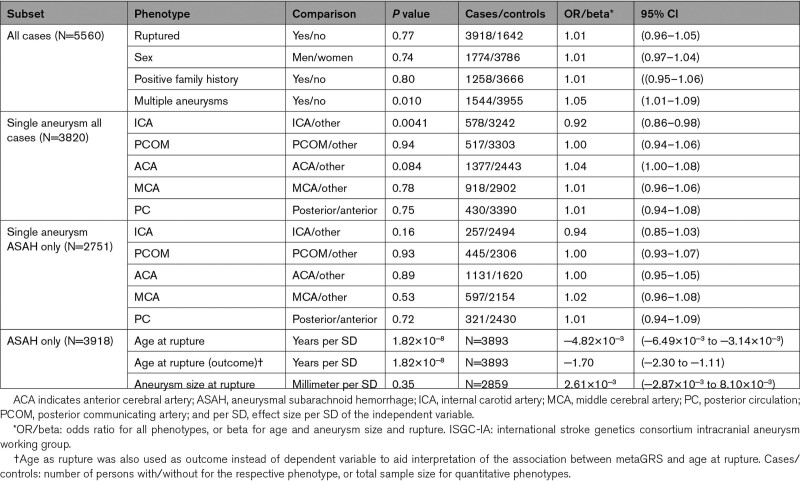
Associations Between metaGRS and Patient Characteristics in the ISGC-IA Phenotype Cohort

In the multivariate model, the association of multiple IAs with metaGRS was not independent of smoking and hypertension (OR=1.03 [95% CI, 0.99–1.08]; *P*=0.16). Upon including smoking and hypertension, the effect of location at the ICA slightly reduced and became nominally statistically significant (OR=0.93 [95% CI, 0.87–0.99]; *P*=0.021), while the association between age at ASAH and metaGRS remained essentially the same (β=−5.2×10^−3^ per year [95% CI, −7.03×10^−3^ to −3.30×10^−3^]; *P*=6.15×10^−8^; Table S13 through S15). Since the mean metaGRS was higher in persons from Finland (Figure S19) we performed the associations analyses on multiple IAs, location at the ICA, or age at ASAH excluding these persons, but the effect sizes remained essentially the same (Table S16).

## Discussion

We created a metaGRS for IA based on GWAS summary statistics for IA and 17 IA-related traits and showed that this metaGRS was predictive of ASAH incidence but not of IA presence. The metaGRS led to only limited improved prediction of ASAH on top of clinical risk factors. We demonstrated that prediction by the metaGRS for ASAH, which disease is seen more often in women than in men,^4,5^ performs better in women than in men. Last, we showed that the metaGRS was higher in patients who suffered ASAH at a younger age and lower in patients with an IA located at the ICA, with both associations being independent of hypertension and smoking.

In a previous study, no association was found between UIA, and genetic risk using a 10-SNP GRS in 109 persons with UIA and 4781 controls.^14^ This may be explained by the low number of patients studied and SNPs included in the GRS. Otherwise, it may be argued that the lack of association is caused by the fact that only UIAs were studied as in our study we were also unable to predict IA presence (combined group of UIA or ASAH) with our metaGRS. However, we think that in our study, we were unable to predict IA presence because many UIAs are likely to be left undetected since UIAs are often incidental findings and therefore have a high chance of not being diagnosed in participants of observational population cohorts as used in our study.^26^ This probably resulted in low statistical power for prediction of UIA alone or in combination with ASAH. In the previous study on 109 persons with UIA and 4781 controls, all participants were systematically screened with for UIAs using brain MRI.^14^ To improve prediction in the future, we recommend confirming absence of IA in controls.

MetaGRSs have been developed for other cardiovascular diseases, including ischemic stroke and coronary artery disease.^9,10^ Here, we found a hazard ratio per SD of metaGRS for prediction of ASAH of 1.34 (95% CI, 1.20–1.51), which was lower than the one previously assessed for coronary artery disease (HR=1.71 [95% CI, 1.68–1.73]) but higher than assessed for ischemic stroke (HR=1.26 [95% CI, 1.22–1.31]).^9,10^ ASAH may be more difficult to predict due to its heterogeneity in characteristics (eg, IA location, size, and rupture risk),^11,12^ and the fact that these characteristics differ between sexes and populations,^27^ necessitating a more personalized approach.

A previous study indicated that persons with IA at the middle cerebral artery had higher genetic risk than persons with IA at other locations, while no associations were found for aneurysmal size at ASAH, patient age at ASAH, or family history of UIA/ASAH.^13,14,28^ We did not replicate the increased genetic load for patients with an IA at the MCA. Since this effect was found in participants from Finland and the Netherlands, and we included additional countries, this might indicate population-dependent heterogeneity. Alternatively, due to the smaller sample size (N=1613) the previous study may have been more sensitive to false positive findings, meaning there is no true effect. Instead, we found a decreased genetic load in patients with an IA at the ICA, which location was not analyzed in the previous study.^13^ Interestingly, IAs at the ICA also have the lowest rupture risk compared to IA at other locations.^29^ This could mean that location-specific rupture risks are in part a downstream result of genetic risk factors, but this remains to be confirmed in future studies.

The predictive performance of the metaGRS was in part captured by the inclusion of clinical risk factors smoking and SBP. This further supports the importance of genetic predisposition for smoking and blood pressure in the risk for ASAH.^2^ This could mean that the remaining added value of the metaGRS is driven by additional genetic causes independent of smoking and SBP, or that the metaGRS better captures lifelong exposure to smoking and SBP than single clinical measurements of these phenotypes.

Prevalence of IA and incidence of ASAH is higher in women than men, in contrast to most other cardiovascular diseases.^5,26^ Here, we found improved prediction of ASAH when the metaGRS was trained and validated in women, and reduced when trained and validated in men. Predictive value of clinical risk factors was also better in women than in men. Sex differences are known in the number and location of IAs, for which characteristics we also showed differences in genetic load.^30^ To understand the difference in genetic mechanisms of IA between men and women future investigations of genetic risk factors for IA and ASAH need to emphasize on sex differences and interactions between genetic variants and sex.

In summary, we developed a metaGRS which showed predictive ability for ASAH but with only limited added value over clinical risk factors. Therefore, there seems to be no place for its use in clinical practice at the moment. However, genetic risk prediction was better in women than in men, warranting further study on the potential of sex-specific disease prediction in combination with assessment of sex-specific genetic causes of IA. The metaGRS was associated with age at ASAH and IA location, showing further evidence for a role of genetic risk in clinical heterogeneity of IA and this heterogeneity should be prioritized in future genetic studies of IA and ASAH.

## Article Information

### Acknowledgments

We thank the ICBP consortium, MEGASTROKE consortium, Trøndelag Health Study (HUNT), and the ISGC for providing summary statistics. This research has been conducted using the UK Biobank Resource under application number 2532.

### Sources of Funding

We acknowledge the support from the Netherlands Cardiovascular Research Initiative: An initiative with support of the Dutch Heart Foundation (CVON2015-08 ERASE), and the European Research Council (ERC) under the European Union’s Horizon 2020 research and innovation program (grant agreement No. 852173). The project was funded in part by NIH grant R35HL161016 and University of Michigan Taubman Institute.

### Disclosures

Dr Veldink has sponsored research agreements with Biogen. Dr Abraham is employed by CSL innovation and received a speaking honorarium for Amgen. Dr Koido is a consultant for Takeda Pharmaceutical Company Limited. Dr Kamatani received speaking honoraria for Astellas Pharma, Chugai Pharmaceutical Co. Ltd, Illumina Japan, Sandoz, and Taisho Pharmaceutical Co. Ltd, and is stockholder of StaGen Co. Ltd. Dr Winsvold is local principal investigator in a study for Lundbeck and gave lectures for Novartis and Lundbeck.

### Supplemental Material

Checklist

Figures S1–S19

Consortium authors

Tables S1–S16

References 31–34

## Supplementary Material


