# Supplementary material for: Genetic Risk Score for Intracranial Aneurysms: Prediction of Subarachnoid Hemorrhage and Role in Clinical Heterogeneity
Source: Stroke. 2023 Jan 19;54(3):810–8. doi: 10.1161/STROKEAHA.122.040715 (PMC9951795; doi:10.1161/STROKEAHA.122.040715)
Supplement: Supplementary file 2 [file str-54-810-s002.pdf]

SUPPLEMENTAL MATERIAL

Genetic risk score for intracranial aneurysms: prediction of subarachnoid hemorrhage and role in clinical heterogeneity

Table of Contents

Supplementary Methods 2

Association data preprocessing 2

Cohort descriptions 2

Genetic correlation 2

Training the trait-level genetic risk scores 2

Association of trait-level GRSs with their respective traits 3

Creating the metaGRS from trait-level GRSs 3

Covariate selection for metaGRS prediction assessment 3

Creating a leave-one-out metaGRS for association with patient characteristics 4

Association between metaGRS and patient characteristics 4

Supplementary Figures 5

Consortium authors 24

HUNT All-In Stroke: 24

CADISP Group 24

International Consortium for Blood Pressure (ICBP): 24

International Headache Genetic Consortium (IHGC): 29

International Stroke Genetics Consortium (ISGC) Intracranial Aneurysm Working Group: 31

Supplementary Methods

Association data preprocessing

For each set of summary statistics obtained from sources described in Supplementary Table 1, the following pre-processing steps were taken. Missing alternate alleles, minor allele frequencies (MAF) and chromosomal positions were annotated from the haplotype reference consortium (HRC) release 1.1 on reference genome GRCh37. If single-nucleotide polymorphism (SNP) beta, standard error (SE), and/or P-value were not available, these were calculated from the available or annotated effect size, SE, P-value, Z-score, sample size, and MAF. Effective sample size and per-SNP sample sizes were used where available.

Cohort descriptions

UK Biobank.[31] This dataset was used to determine the optimal trait-level GRSs, and to combine trait-level GRSs into a metaGRS. Persons with an unruptured intracranial aneurysm (UIA) or aneurysmal subarachnoid hemorrhage (ASAH), both assessed by ICD-10 codes, were selected as intracranial aneurysm (IA) cases. Persons with a non-aneurysmal subarachnoid hemorrhage, with another ancestral background than white British, or with a diagnosis of autosomal dominant polycystic kidney disease, Ehlers-Danlos disease or Marfan syndrome, were excluded. SNPs with MAF < 0.005 or imputation INFO-score < 0.60 were excluded. This resulted in 1,161 intracranial aneurysm (IA) patients, of which 959 had an ASAH, 202 with UIA, and 408,553 controls.

HUNT study. The Trøndelag Health Study (HUNT) is a collaboration between HUNT Research Centre (Faculty of Medicine and Health Sciences, Norwegian University of Science and Technology NTNU), Trøndelag County Council, Central Norway Regional Health Authority, and the Norwegian Institute of Public Health. The genotyping was financed by the National Institute of health (NIH), University of Michigan, The Norwegian Research council, and Central Norway Regional Health Authority and the Faculty of Medicine and Health Sciences, Norwegian University of Science and Technology (NTNU). The genotype quality control and imputation has been conducted by the K.G. Jebsen center for genetic epidemiology, Department of public health and nursing, Faculty of medicine and health sciences, Norwegian University of Science and Technology (NTNU).

This dataset was used to assess predictive performance of the metaGRS for ASAH hazard and IA presence. Detailed information about inclusion criteria, diagnosis and genotyping has been described before.[32,33] In brief, UIA and ASAH diagnosis was done by ICD-10 codes I67.1 and I60. A total of 828 IA patients, of which 318 with ASAH, and 68,568 without any IA were included. For the analyses studying ASAH, the UIA cases were included as controls, resulting in 69,078 controls. Genotyping was done using Illumina HumanCoreExome platforms.

Phenotype cohort. From a subset of the largest GWAS on IA, detailed phenotype information was obtained.[15] The phenotype cohort was used to assess which phenotypes were associated with genetic predisposition for IA. Persons without genetic data were excluded. Phenotypic information was obtained in 5,560 IA patients of which 3,916 with ASAH from 18 European cohorts. In 1,544 patients multiple IAs were found. Phenotypes were obtained from imaging data or surgical exploration.

Genetic correlation

To select traits to include in elastic net regression, we calculated genetic correlation between IA and the respective trait using linkage disequilibrium score regression (LDSC).[34] HapMap v3 linkage disequilibrium (LD) scores were used as reference panel. Only SNPs with minor allele frequency > 5% were included, and an LD window of 200 kb was selected. Traits with a genetic correlation P-value < 0.05 were selected to create a trait-level GRS.

Training the trait-level genetic risk scores

Eleven trait-level GRSs were calculated for each trait according to three methods: LD-based clumping with 9 LD thresholds, summary statistics-based best linear unbiased predictor (s-BLUP)[35] and summary statistics-based bayes R (s-Bayes R)[8]. No P-value threshold was used to select SNPs for any of the models. Control samples included in the largest IA GWAS[2] (stratum sNL2) were used as LD reference panel. Clumping was done to exclude LD-correlated SNPs, while retaining the SNPs with lowest P-values. This was done using plink v1.9.

LD r-squared thresholds ranging from 0.1 to 0.9 (interval 0.1) were used. s-BLUP scales SNP weights to provide LD-adjusted linear predictors. s-BLUP lambda was calculated by dividing the number of SNPs in common between summary statistics and LD reference panel by the following: the inverse of the SNP-based heritability estimate as assessed by LDSC, minus 1. For heritability estimation, the same SNPs and settings were used as for genetic correlation estimation. s-Bayes R uses a Bayesian approach to calculate LD-adjusted SNP effects suitable for genetic risk prediction. For s-Bayes R, a sparse LD matrix was created with SNPs in common between the LD reference panel and summary statistics. An interpolated genetic map with centimorgan positions was used (ftp://ftp.1000genomes.ebi.ac.uk/vol1/ftp/technical/working/20130507_omni_recombination_rates/). SNP weights were then calculated with a pi vector of 0.95, 0.02, 0.02 and 0.01 and a gamma vector of 0, 1, 10 and 100, while allowing for unscaled genotypes. A burn-in of 10,000 iterations and total chain of 20,000 iterations were selected.

For each of the 11 models (9 clumping thresholds, s-BLUP, and s-Bayes R), model performance was assessed in the UK Biobank cohort by logistic regression in R (function *glm*), using sex (in model analyzing whole cohort) and 10 genetic principal components as covariates. The GRS with the highest Nagelkerke pseudo R-squared was selected for elastic net regression, leaving each trait with three optimal trait-GRS models: one for the whole cohort, one for men only and one for women only.

Association of trait-level GRSs with their respective traits

We assessed whether trait-level GRSs were associated with their respective traits in the UK Biobank. Trait-level SNP weights as included in the metaGRS were used. Traits included in the metaGRS, for which a representative phenotype was available in the UK Biobank, with at least 100 cases (for binary traits) were selected for this analysis (Supplementary Table 6). ICD-10 codes for hospitalization, or death record were used for binary traits. Individual-level trait-level GRSs were calculated using *plink (v2.0)* function *--score*, only including SNPs with INFO-score above 0.8. Each trait-level GRS was transformed to zero mean, unit variance. For binary traits, a logistic regression with trait-level GRS as independent variable, respective trait as dependent variable, and sex and age as covariates was performed in R. A model excluding the trait-level GRS was used to assess the improvement of the area under the receiver operator characteristic curve upon adding the trait-level GRS. For quantitative traits a linear regression was performed using the same variables. Added value of the trait-level GRS was assessed by comparing r-squared value between the models including and excluding the trait-level GRS. For age at menarche, only women were included, and sex was therefore not included as covariate.

Creating the metaGRS from trait-level GRSs

Individual level trait-level GRSs (of the optimal trait-level GRSs) were calculated using *plink (v1.9)* function *--score* in the UK Biobank cohort, only including SNPs with INFO-score above 0.8. All trait-level GRSs were then standardized to mean 0 and standard deviation 1. Elastic net regression was performed on all trait-level GRSs combined using R function *glmnet* using covariates sex (in model analyzing whole cohort) and 10 genetic principal components. Ten-fold cross-validation was performed to optimize the area under the curve and thereby obtain the trait-level GRS elastic net weights. For each trait, per-SNP weights from the trait-level GRS models were multiplied by the elastic net weight for that trait and divided by the population GRS standard deviation for that trait in the UK Biobank cohort prior to standardizing. These per-SNP weights were summed over traits, creating per-SNP weights of the metaGRS. The IA trait-level SNP weights after elastic net regression were also used as a IA-only GRS to compare metaGRS and IA-only GRS performance.

Covariate selection for metaGRS prediction assessment

Variables to include in the prediction assessment model were selected by logistic regression (function *glm* in R) with IA as outcome in the UK Biobank. Variables with a statistically significant effect in the UK Biobank (P-value < 0.05), while correcting for sex and age (except when testing these), were included in the model. Variables tested were: sex, age, systolic blood pressure, diastolic blood pressure, alcoholic drinks per week, average smoking packs per day since age 16. Alcoholic drinks per week was not statistically significant as predictor, and diastolic blood pressure was excluded because of the strong correlation with, but less statistically significant effect than, systolic blood pressure. For each variable, we determined whether to use it as linear predictor, cubic spline, or polynomial spline, by optimizing the Akaike information criterion in a logistic regression model with IA as outcome, the respective variable as predictor, and covariates age and sex. We varied splines from 3-10 knots. Age, smoking pack years proportional to lifespan, and SBP were used as polynomial spline with 3 knots, while metaGRS was used as a linear predictor.

Creating a leave-one-out metaGRS for association with patient characteristics

To account for sample overlap between IA GWAS and the phenotype cohort, nine sets of IA GWAS summary statistics were created excluding each stratum in a leave-one-out manner. Stratum sUK, including the UK Biobank, was left out in every set due to overlap with the metaGRS training set (as this stratum was also left out when creating the main metaGRS used for prediction of ASAH hazard and IA presence). Nine metaGRS versions were created by elastic net regression using each leave-one-out IA GWAS once, selecting the same optimal trait-level GRSs (LD clumping, s-Bayes R, or s-BLUP) as used for the main metaGRS. Individual-level GRS of all patients were calculated with plink option *--score* using the per-SNP weights of the metaGRS leaving out the patients' respective stratum, as SNP scores.

Calculating predicted cumulative incidence

Cox regression was performed using R function *coxph*. Baseline hazard at age 75 was calculated using R function *basehaz*. Predicted cumulative incidence of a person of age 75 with varying sex and metaGRS was calculated by multiplying the baseline hazard with natural exponentiation of the log-hazard ratio obtained by the *predict* function.

Association between metaGRS and patient characteristics

We calculated the effect of various phenotypes (hypertension, smoking status, IA multiplicity, rupture status, age at ASAH, aneurysmal size at rupture, family history, and IA locations) on the metaGRS. To determine the effect of age at ASAH and aneurysmal size at rupture, only ASAH patients were included. Persons with an aneurysmal size below 1.5 times the interquartile range lower than the first quartile, or above 1.5 times the interquartile range higher than the third quartile were considered outliers and excluded from the aneurysmal size analysis. When assessing the effect of IA location, only patients with a single IA were included. For each phenotype, a generalized linear model was fitted with metaGRS as dependent variable, the phenotype of interest as independent variable, and cohort and sex as covariates. To obtain an interpretable effect size of metaGRS on age at ASAH, an additional analysis was done with age at ASAH as outcome and metaGRS as independent variable. Effect size of IA phenotypes on the metaGRS were transformed to unit variance by dividing the effect size by the standard deviation of the metaGRS among included samples. Odds ratios were then calculated for binary phenotypes by natural exponentiation.

To test whether the effect of a phenotype on the metaGRS remained when including other phenotypes, we used a stepwise selection model (R function *stepAIC*). One model was used for each subset (all cases, single IA cases, and ASAH cases). Each model included sex, hypertension, smoking status, and cohort. Depending on the subset of cases, the model could also include IA multiplicity, rupture status, family history, and IA location at the ICA.

Supplementary Figures

Supplementary Figure 1. Predictive performance for aneurysmal subarachnoid hemorrhage of the metaGRS in women and men, alone. A) metaGRS trained using an elastic net regression in women included the UK Biobank, and validated in women included in the HUNT study. B) The same as panel A, but trained and validated in men.

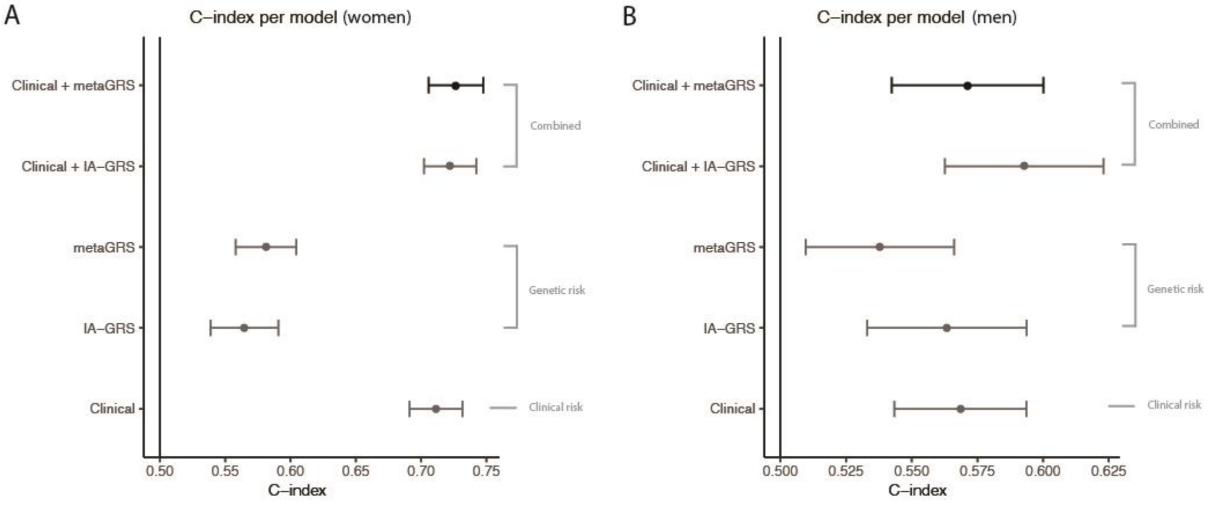

Supplementary Figure 2. Predictive performance for intracranial aneurysm presence of the metaGRS. metaGRS trained using an elastic net regression in men and women combined in the UK Biobank, and validated in the HUNT study. Reference: prediction model including age and sex only. Clinical: model including age + sex + systolic blood pressure (SBP) + smoking packs per day. metaGRS: model including age + sex + metaGRS. The models above the dashed line each leave out one variable from the full model which includes age + sex + SBP + smoking packs per day + metaGRS. Error bars denote 95% confidence interval (CI_{95}). AUC: area under the receiver operator characteristic curve.

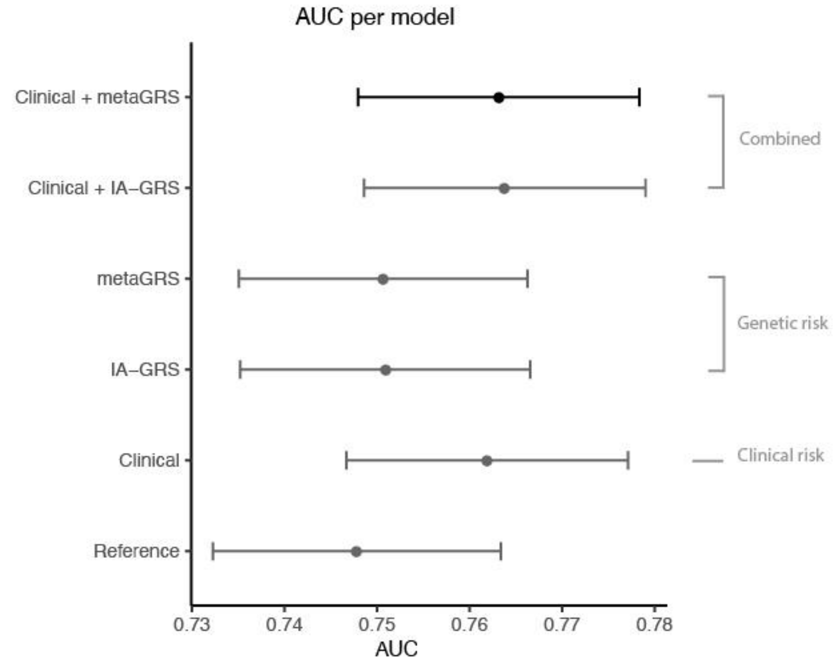

Supplementary Figure 3. Association between metaGRS and intracranial aneurysm (IA, either ruptured or unruptured) location at the internal carotid artery (ICA) versus other locations. A) Violin plot of the distribution of metaGRS in the phenotype cohort among persons with an IA at the ICA and the remaining group. Horizontal lines denote mean and mean ± 1 standard deviation. B) Box plots showing the distribution in each sub-cohort within the phenotype cohort. Boxes contain 25th to 75th percentile and denote the median with a horizontal line. Whiskers denote smallest value greater than 1.5 times the interquartile range below the 25th percentile, and largest value smaller than 1.5 times the interquartile range above the 75th percentile.

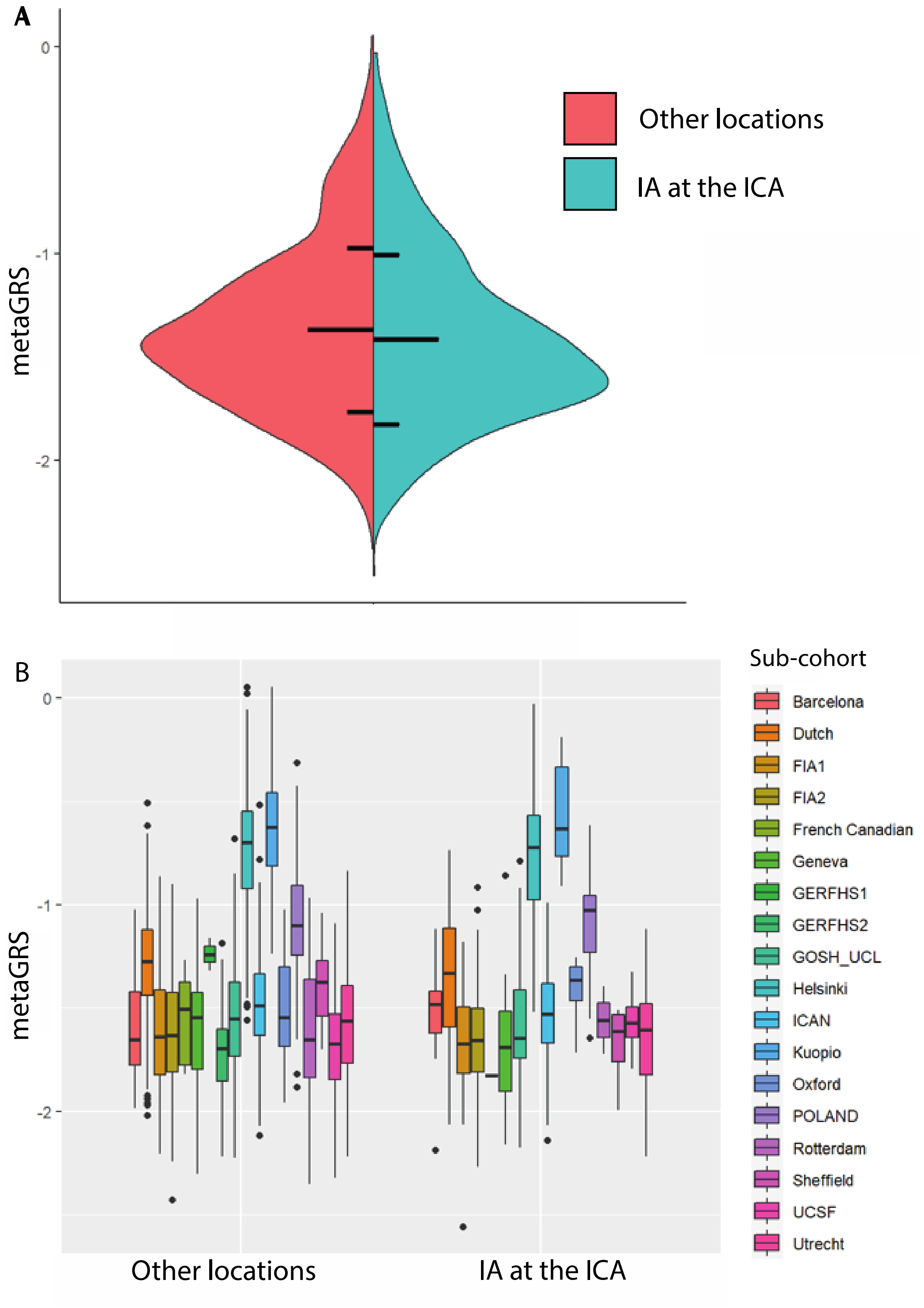

Supplementary Figure 4. Association between metaGRS and intracranial aneurysm (IA, either ruptured or unruptured) location at the posterior communicating artery (PCOM) versus other locations. A) Violin plot of the distribution of metaGRS in the phenotype cohort among persons with an IA at the PCOM and the remaining group. Horizontal lines denote mean and mean ± 1 standard deviation. B) Box plots showing the distribution in each sub-cohort within the phenotype cohort. Boxes contain 25th to 75th percentile and denote the median with a horizontal line. Whiskers denote smallest value greater than 1.5 times the interquartile range below the 25th percentile, and largest value smaller than 1.5 times the interquartile range above the 75th percentile.

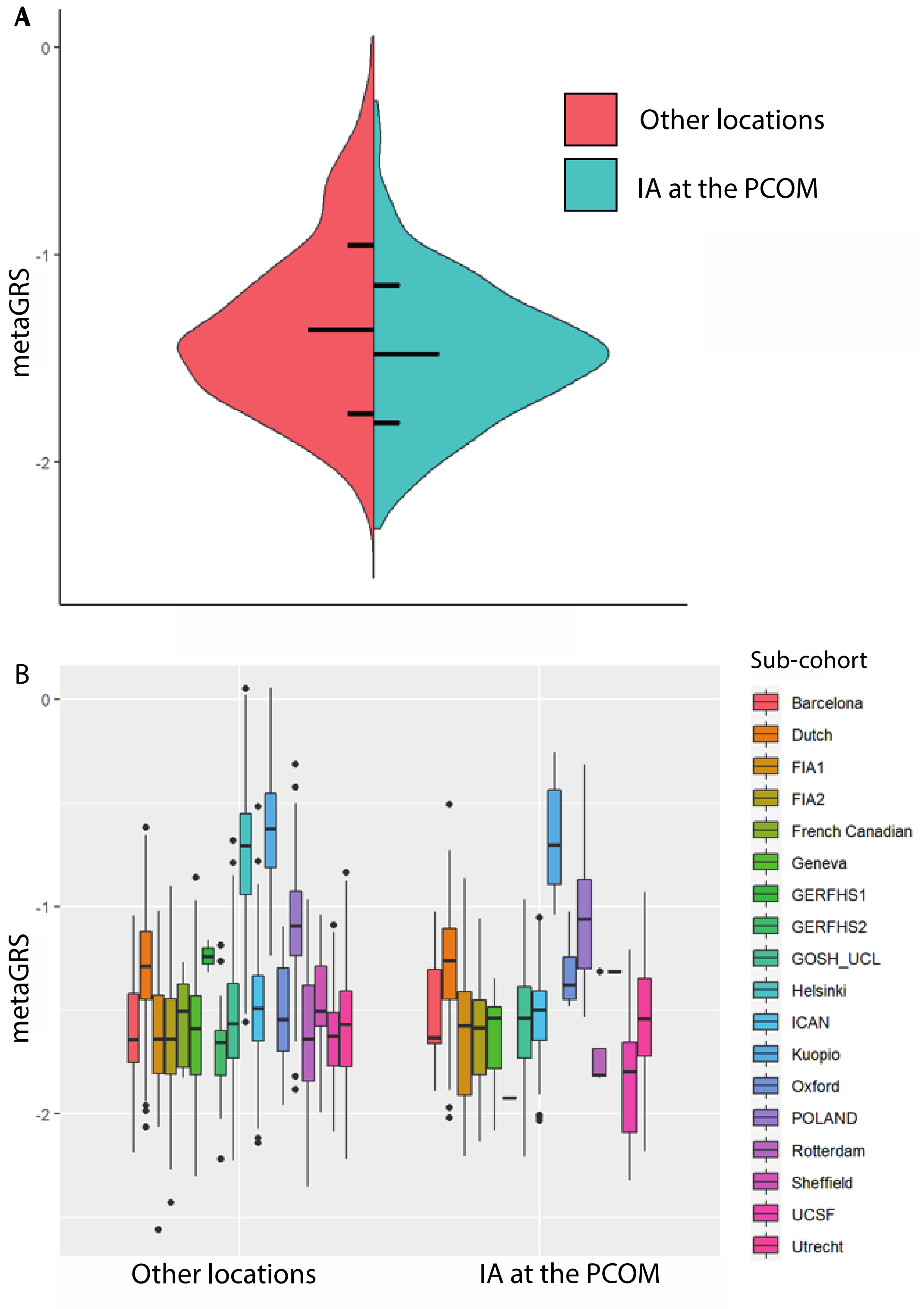

Supplementary Figure 5. Association between metaGRS and intracranial aneurysm (IA, either ruptured or unruptured) location at the anterior cerebral arteries (ACA) versus other locations. A) Violin plot of the distribution of metaGRS in the phenotype cohort among persons with an IA at the ACA and the remaining group. Horizontal lines denote mean and mean ± 1 standard deviation. B) Box plots showing the distribution in each sub-cohort within the phenotype cohort. Boxes contain 25th to 75th percentile and denote the median with a horizontal line. Whiskers denote smallest value greater than 1.5 times the interquartile range below the 25th percentile, and largest value smaller than 1.5 times the interquartile range above the 75th percentile.

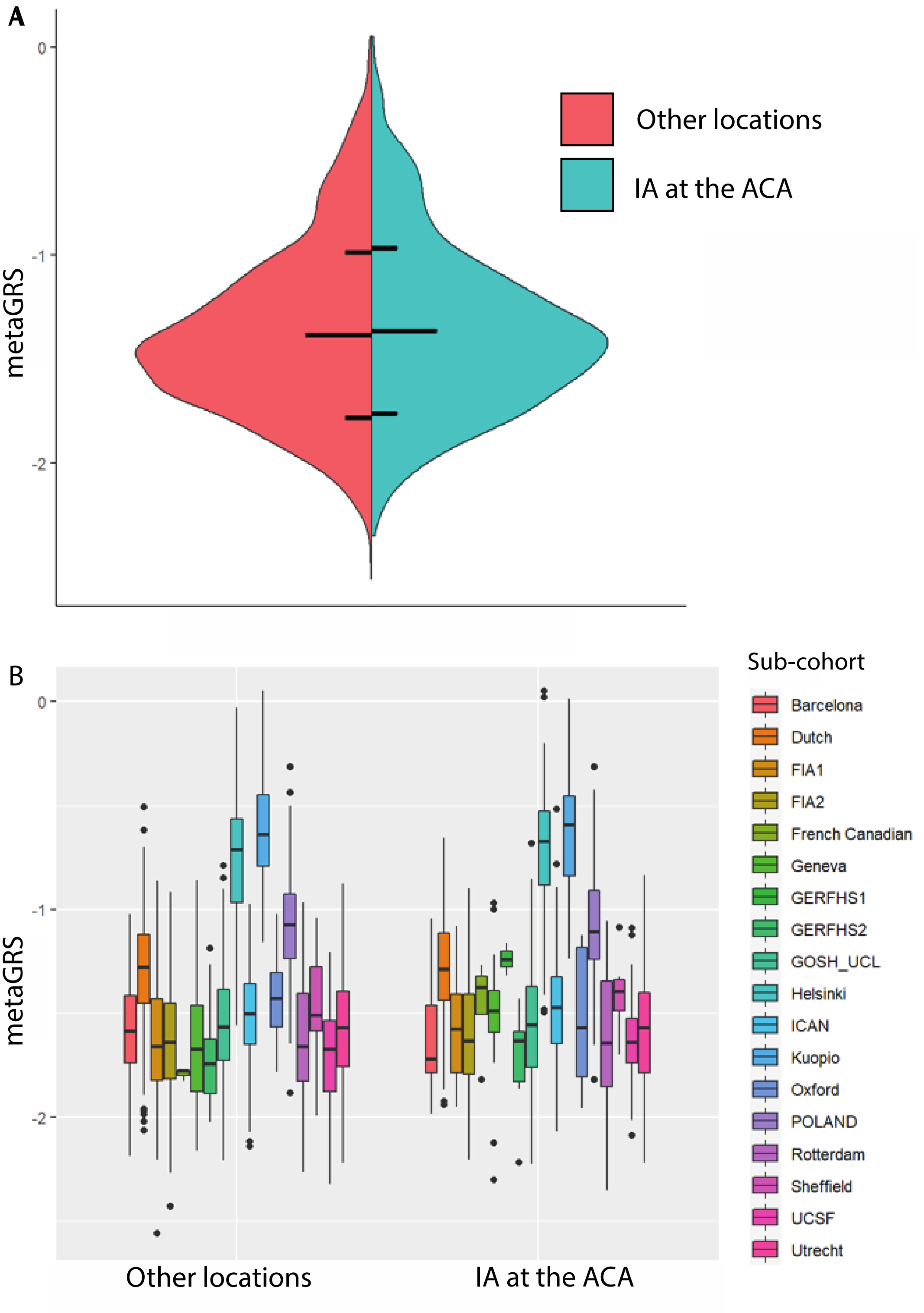

Supplementary Figure 6. Association between metaGRS and intracranial aneurysm (IA, either ruptured or unruptured) location at the middle cerebral artery (MCA) versus other locations. A) Violin plot of the distribution of metaGRS in the phenotype cohort among persons with an IA at the MCA and the remaining group. Horizontal lines denote mean and mean ± 1 standard deviation. B) Box plots showing the distribution in each sub-cohort within the phenotype cohort. Boxes contain 25th to 75th percentile and denote the median with a horizontal line. Whiskers denote smallest value greater than 1.5 times the interquartile range below the 25th percentile, and largest value smaller than 1.5 times the interquartile range above the 75th percentile.

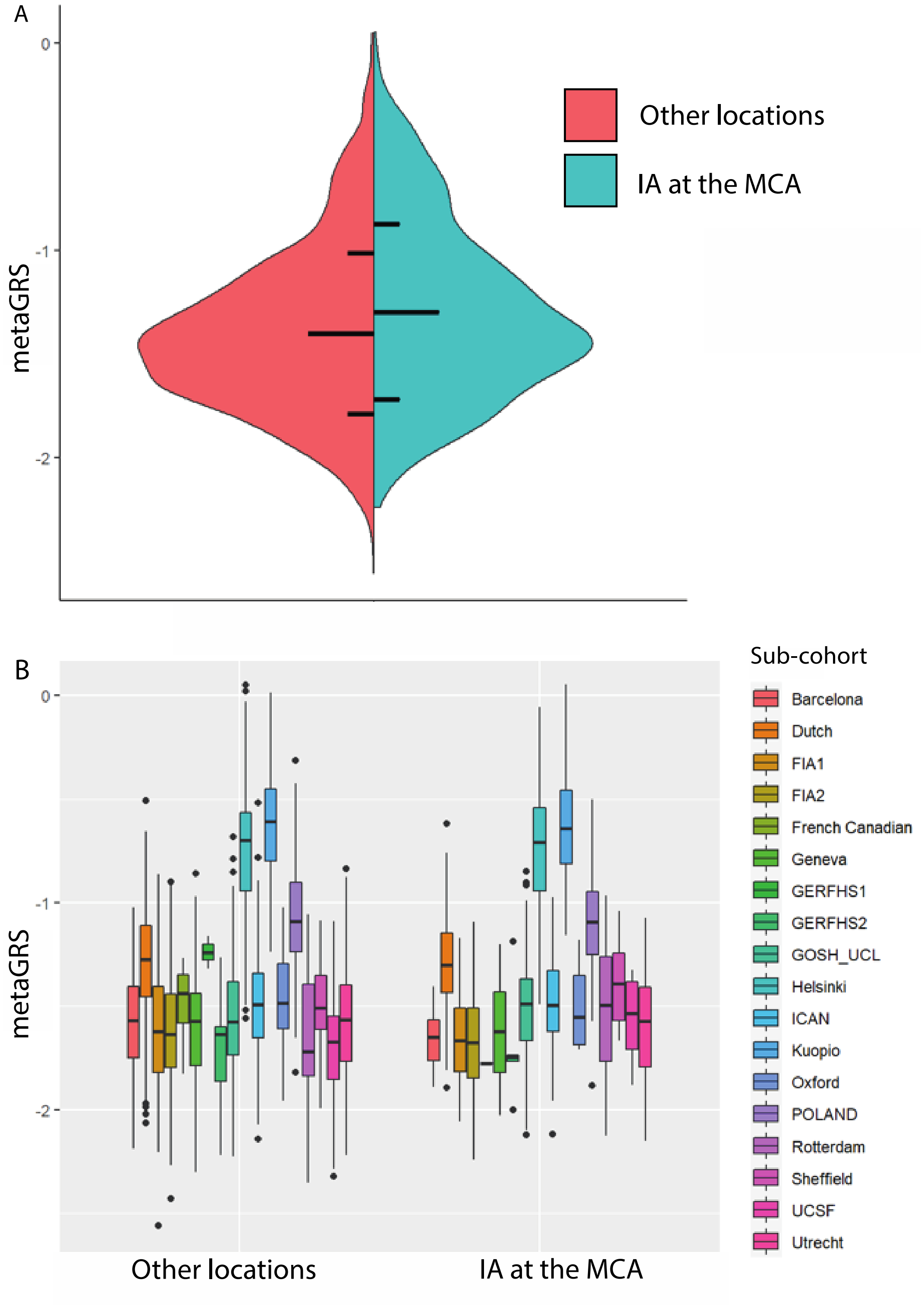

Supplementary Figure 7. Association between metaGRS and intracranial aneurysm (IA, either ruptured or unruptured) location at the posterior circulation arteries (PC) versus other locations. A) Violin plot of the distribution of metaGRS in the phenotype cohort among persons with an IA at the PC and the remaining group. Horizontal lines denote mean and mean ± 1 standard deviation. B) Box plots showing the distribution in each sub-cohort within the phenotype cohort. Boxes contain 25th to 75th percentile and denote the median with a horizontal line. Whiskers denote smallest value greater than 1.5 times the interquartile range below the 25th percentile, and largest value smaller than 1.5 times the interquartile range above the 75th percentile.

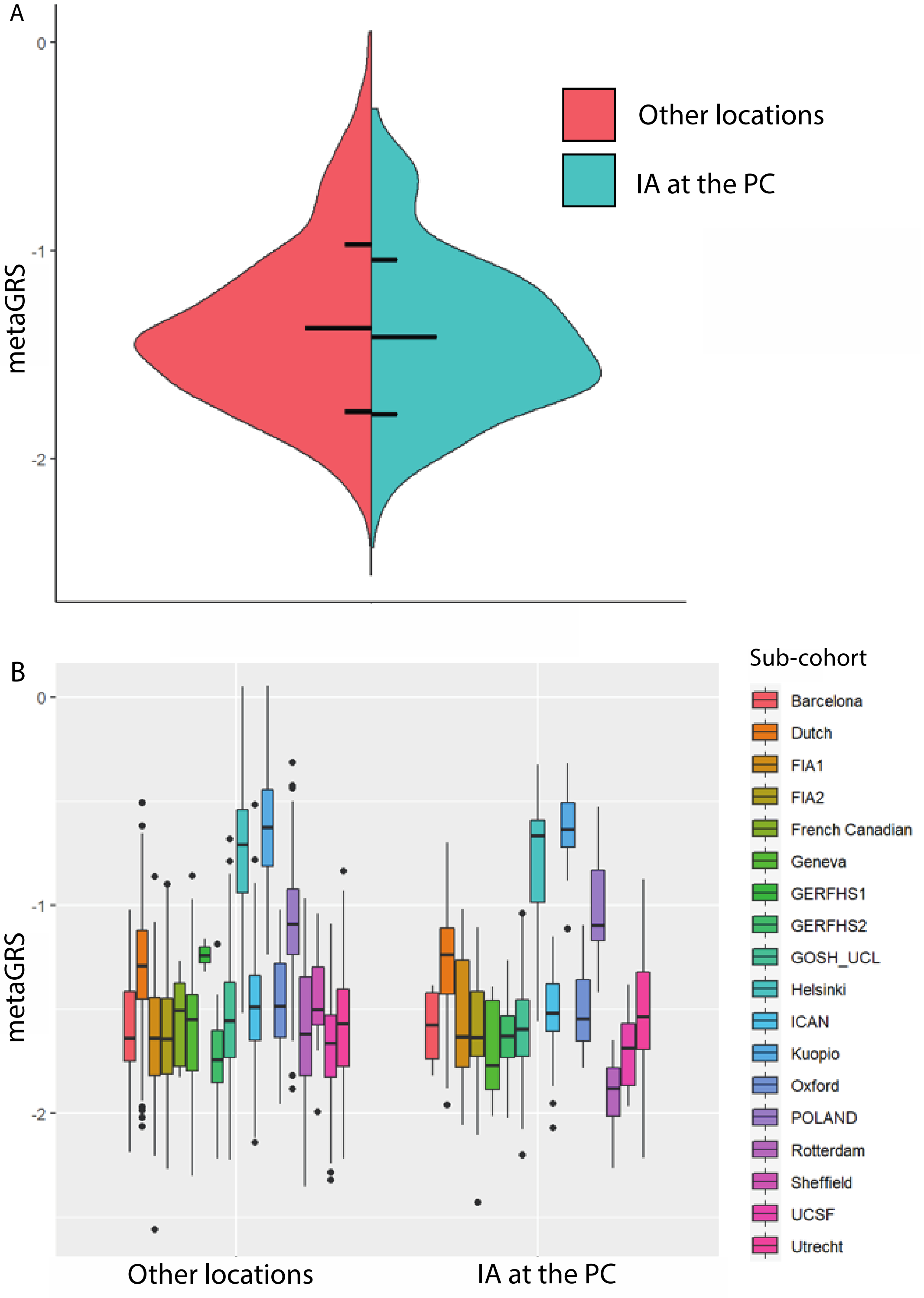

Supplementary Figure 8. Association between metaGRS and aneurysmal subarachnoid hemorrhage (ASAH) from an intracranial aneurysm (IA) at the internal carotid artery (ICA) versus other locations. A) Violin plot of the distribution of metaGRS in the phenotype cohort among persons with an ASAH at the ICA and the remaining group. Horizontal lines denote mean and mean ± 1 standard deviation. B) Box plots showing the distribution in each sub-cohort within the phenotype cohort. Boxes contain 25th to 75th percentile and denote the median with a horizontal line. Whiskers denote smallest value greater than 1.5 times the interquartile range below the 25th percentile, and largest value smaller than 1.5 times the interquartile range above the 75th percentile.

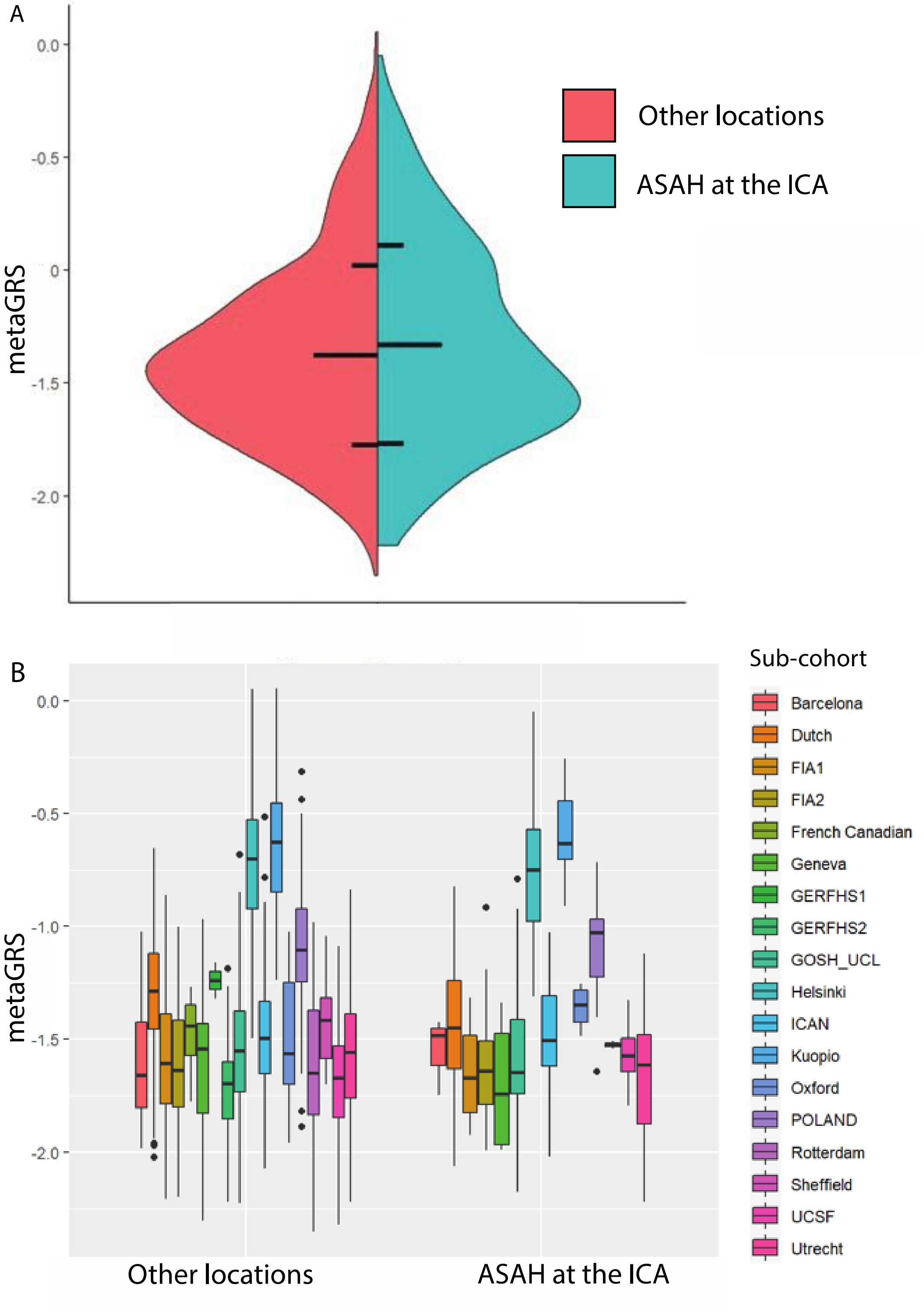

Supplementary Figure 9. Association between metaGRS and aneurysmal subarachnoid hemorrhage (ASAH) from an intracranial aneurysm (IA) at the posterior communicating artery (PCOM) versus other locations. A) Violin plot of the distribution of metaGRS in the phenotype cohort among persons with an ASAH at the PCOM and the remaining group. Horizontal lines denote mean and mean ± 1 standard deviation. B) Box plots showing the distribution in each sub-cohort within the phenotype cohort. Boxes contain 25th to 75th percentile and denote the median with a horizontal line. Whiskers denote smallest value greater than 1.5 times the interquartile range below the 25th percentile, and largest value smaller than 1.5 times the interquartile range above the 75th percentile.

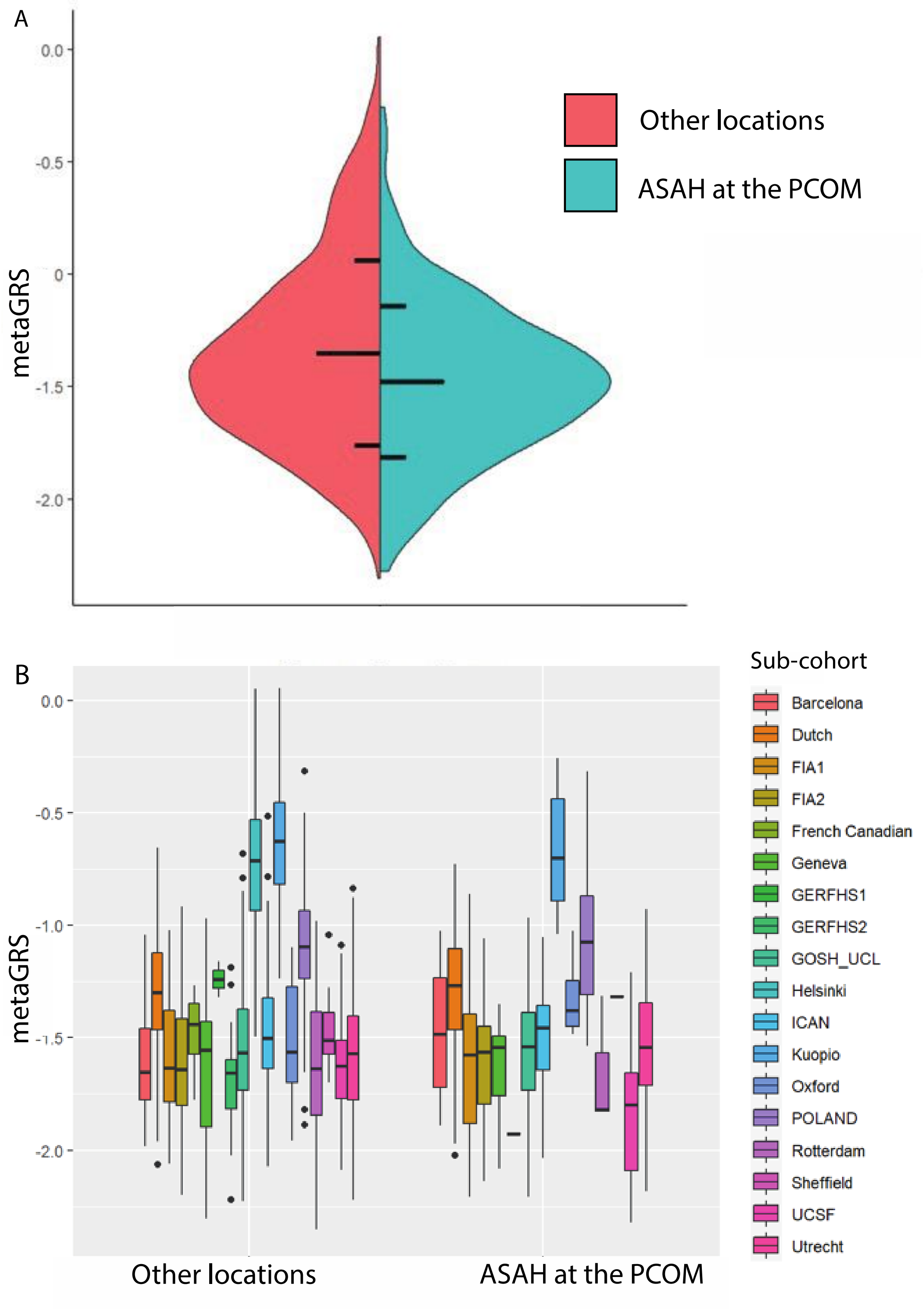

Supplementary Figure 10. Association between metaGRS and aneurysmal subarachnoid hemorrhage (ASAH) from an intracranial aneurysm (IA) at the anterior cerebral arteries (ACA) versus other locations. A) Violin plot of the distribution of metaGRS in the phenotype cohort among persons with an ASAH at the ACA and the remaining group. Horizontal lines denote mean and mean ± 1 standard deviation. B) Box plots showing the distribution in each sub-cohort within the phenotype cohort. Boxes contain 25th to 75th percentile and denote the median with a horizontal line. Whiskers denote smallest value greater than 1.5 times the interquartile range below the 25th percentile, and largest value smaller than 1.5 times the interquartile range above the 75th percentile.

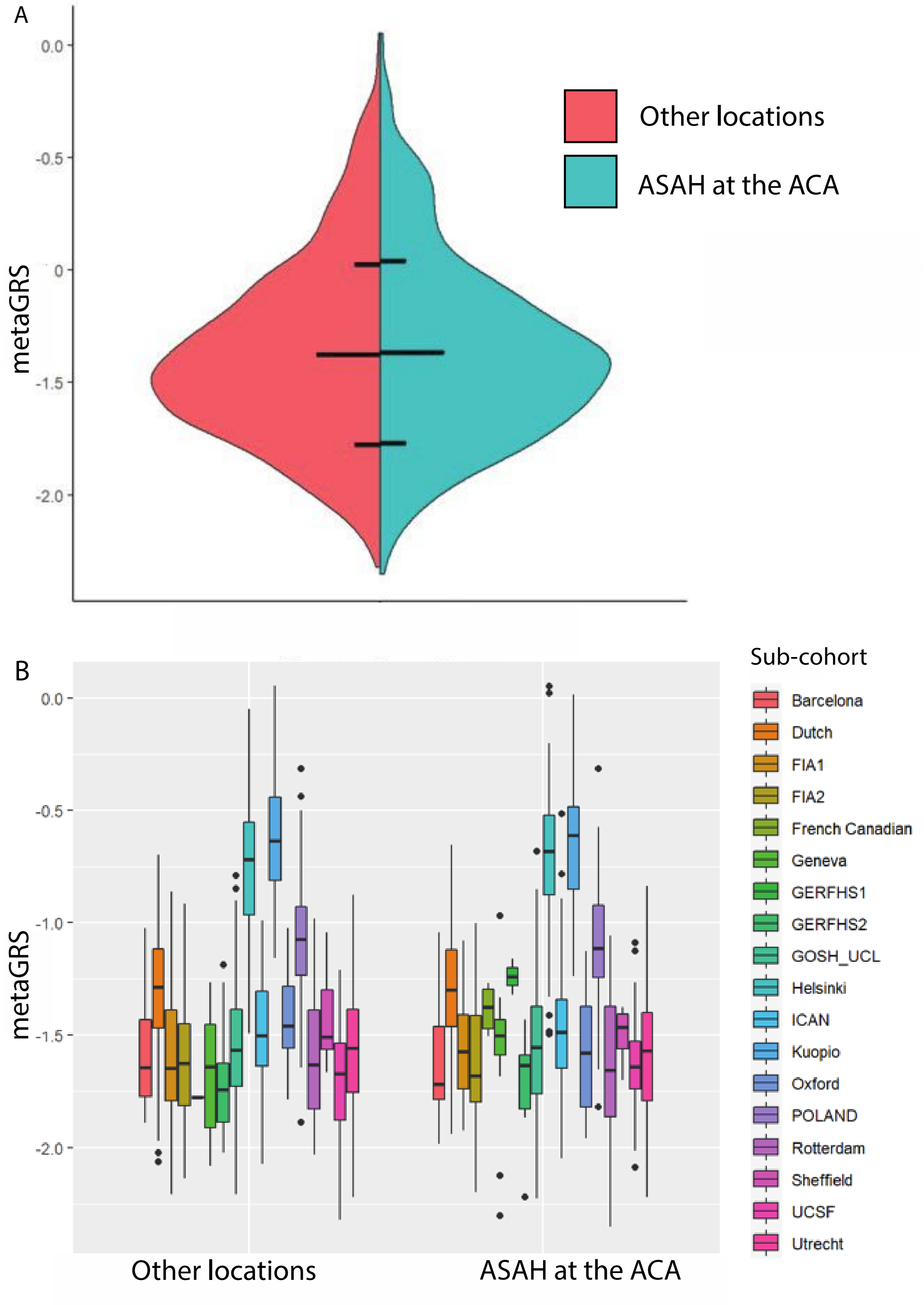

Supplementary Figure 11. Association between metaGRS and aneurysmal subarachnoid hemorrhage (ASAH) from an intracranial aneurysm (IA) at the middle cerebral artery (MCA) versus other locations. A) Violin plot of the distribution of metaGRS in the phenotype cohort among persons with an ASAH at the MCA and the remaining group. Horizontal lines denote mean and mean ± 1 standard deviation. B) Box plots showing the distribution in each sub-cohort within the phenotype cohort. Boxes contain 25th to 75th percentile and denote the median with a horizontal line. Whiskers denote smallest value greater than 1.5 times the interquartile range below the 25th percentile, and largest value smaller than 1.5 times the interquartile range above the 75th percentile.

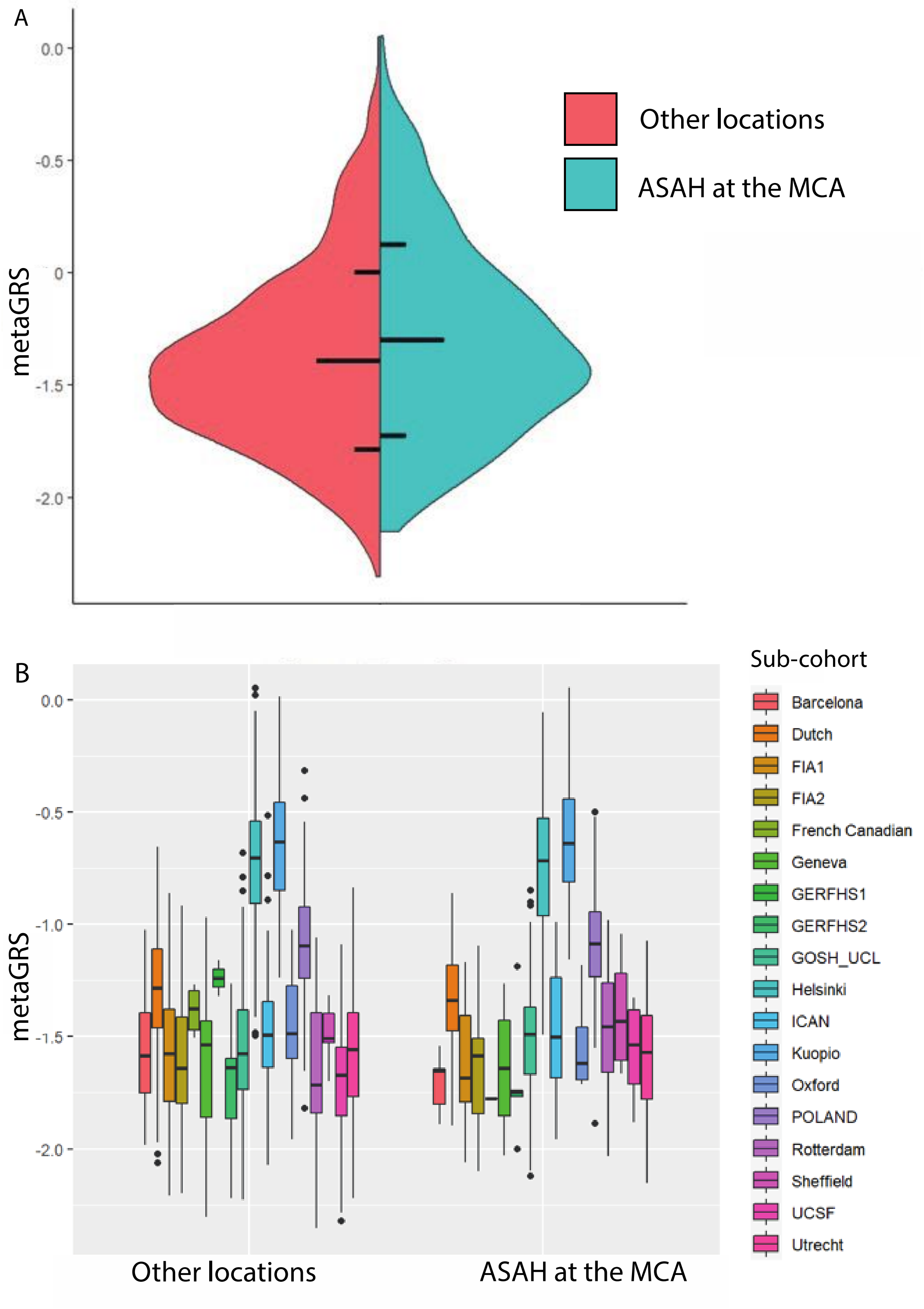

Supplementary Figure 12. Association between metaGRS and aneurysmal subarachnoid hemorrhage (ASAH) from an intracranial aneurysm (IA) at the posterior circulation arteries (PC) versus other locations. A) Violin plot of the distribution of metaGRS in the phenotype cohort among persons with an ASAH at the PC and the remaining group. Horizontal lines denote mean and mean ± 1 standard deviation. B) Box plots showing the distribution in each sub-cohort within the phenotype cohort. Boxes contain 25th to 75th percentile and denote the median with a horizontal line. Whiskers denote smallest value greater than 1.5 times the interquartile range below the 25th percentile, and largest value smaller than 1.5 times the interquartile range above the 75th percentile.

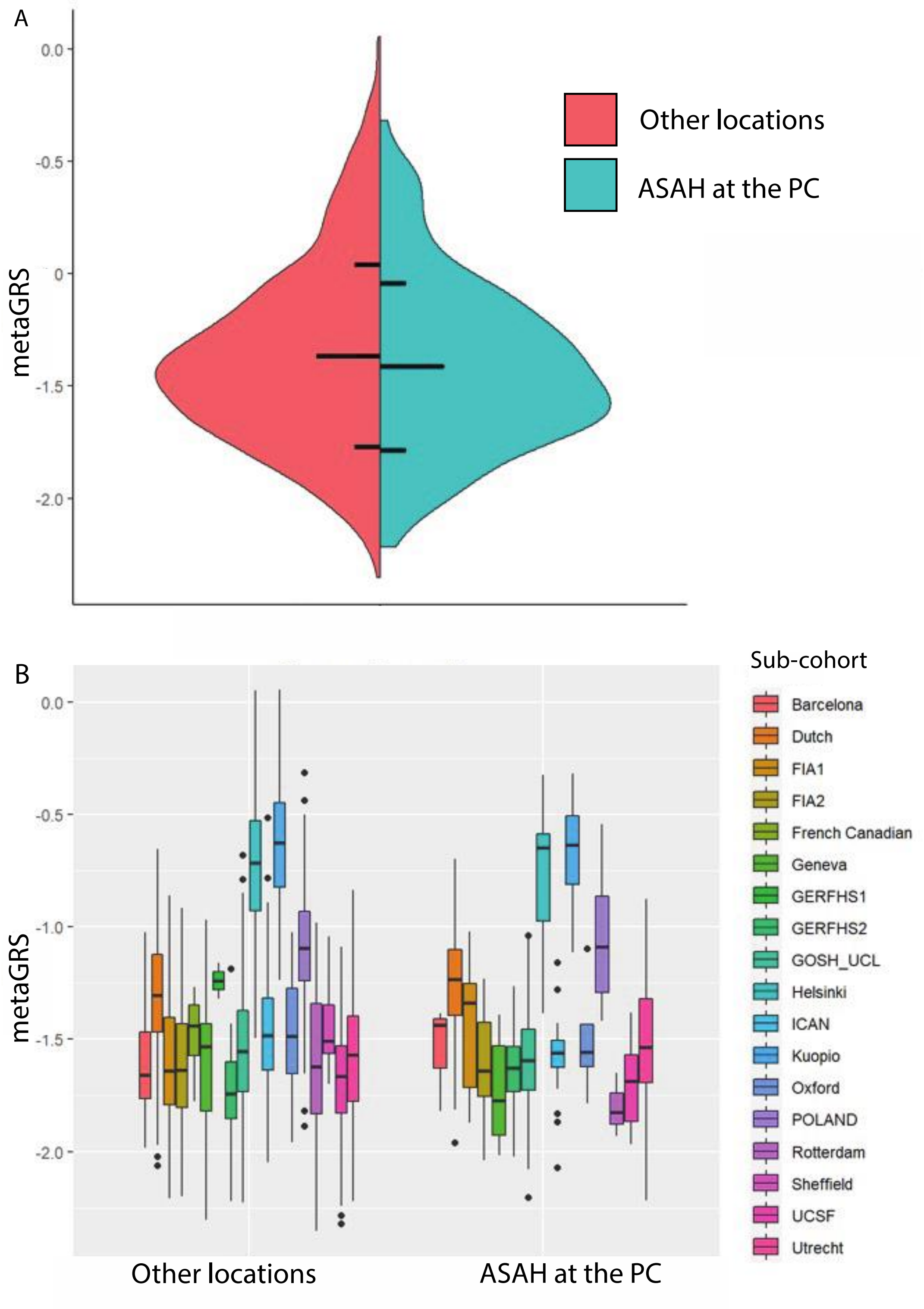

Supplementary Figure 13. Association between metaGRS and sex among persons with an intracranial aneurysm (IA, either ruptured or unruptured). A) Violin plot of the distribution of metaGRS in the phenotype cohort stratified by women and men. Horizontal lines denote mean and mean ± 1 standard deviation. B) Box plots showing the distribution in each sub-cohort within the phenotype cohort. Boxes contain 25th to 75th percentile and denote the median with a horizontal line. Whiskers denote smallest value greater than 1.5 times the interquartile range below the 25th percentile, and largest value smaller than 1.5 times the interquartile range above the 75th percentile.

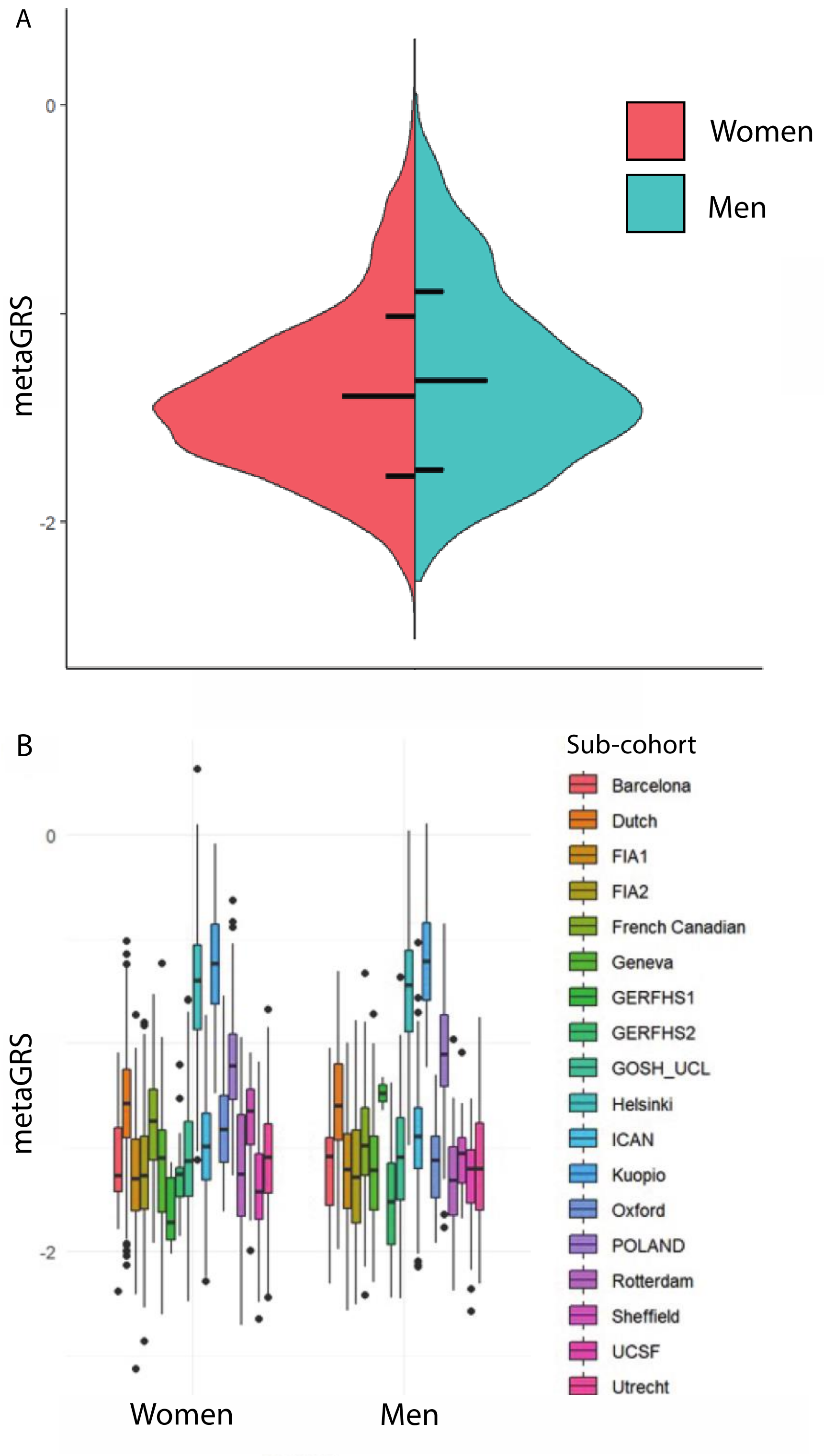

Supplementary Figure 14. Association between metaGRS and family history among persons with an intracranial aneurysm (IA, either ruptured or unruptured). A) Violin plot of the distribution of metaGRS in the phenotype cohort stratified by family history of IA. Horizontal lines denote mean and mean ± 1 standard deviation. B) Box plots showing the distribution in each sub-cohort within the phenotype cohort. Boxes contain 25th to 75th percentile and denote the median with a horizontal line. Whiskers denote smallest value greater than 1.5 times the interquartile range below the 25th percentile, and largest value smaller than 1.5 times the interquartile range above the 75th percentile.

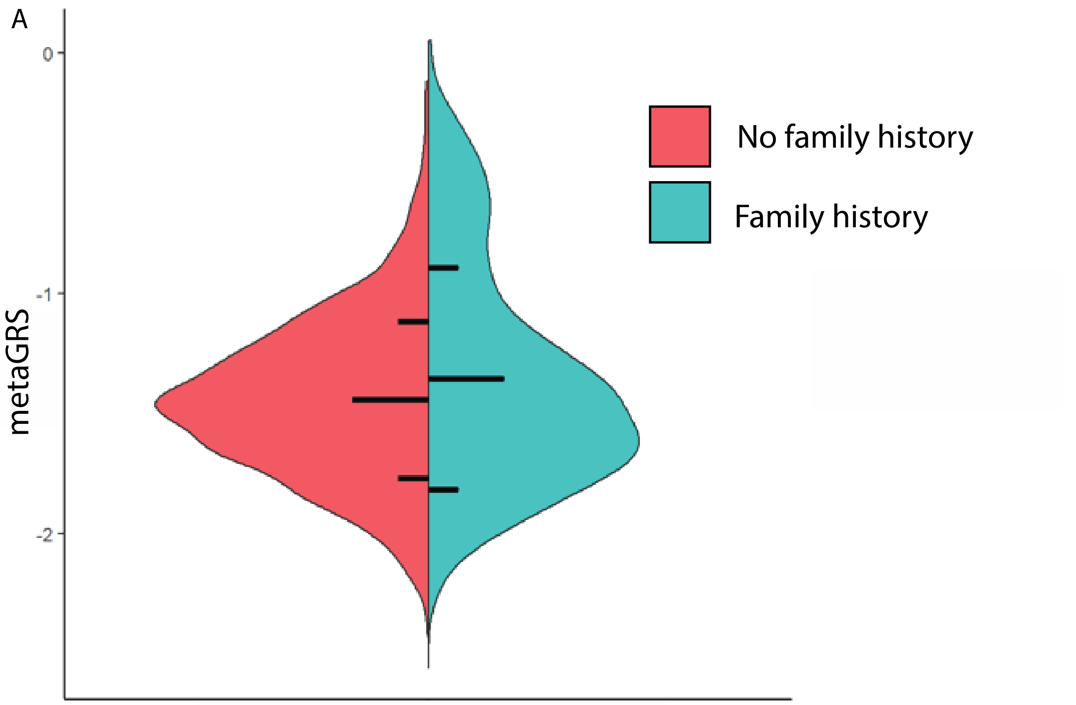

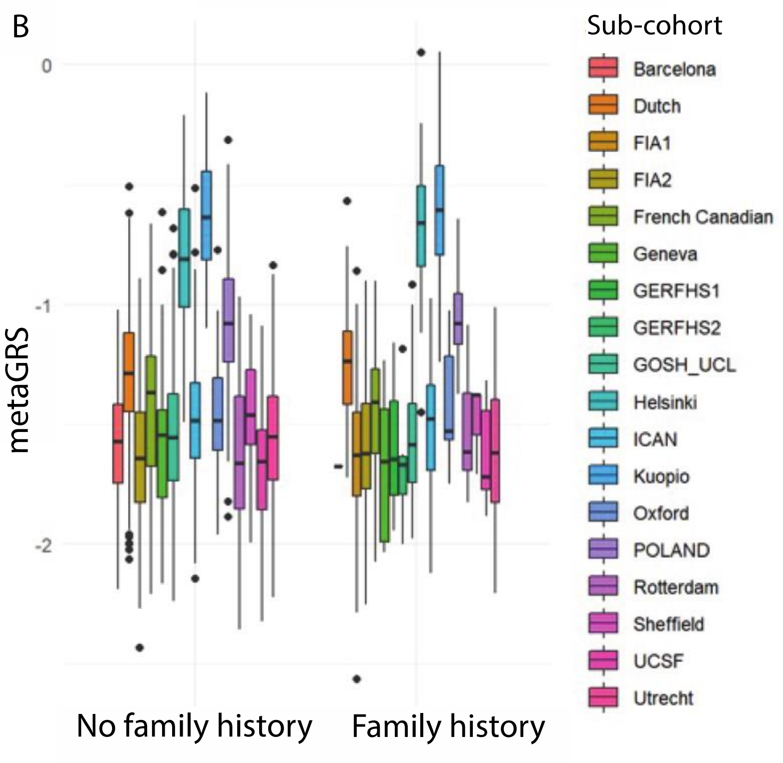

Supplementary Figure 15. Association between metaGRS and rupture status of an intracranial aneurysm (IA). A) Violin plot of the distribution of metaGRS in the phenotype cohort stratified by rupture status (unruptured IA, UIA versus aneurysmal subarachnoid hemorrhage, ASAH). Horizontal lines denote mean and mean ± 1 standard deviation. B) Box plots showing the distribution in each sub-cohort within the phenotype cohort. Boxes contain 25th to 75th percentile and denote the median with a horizontal line. Whiskers denote smallest value greater than 1.5 times the interquartile range below the 25th percentile, and largest value smaller than 1.5 times the interquartile range above the 75th percentile.

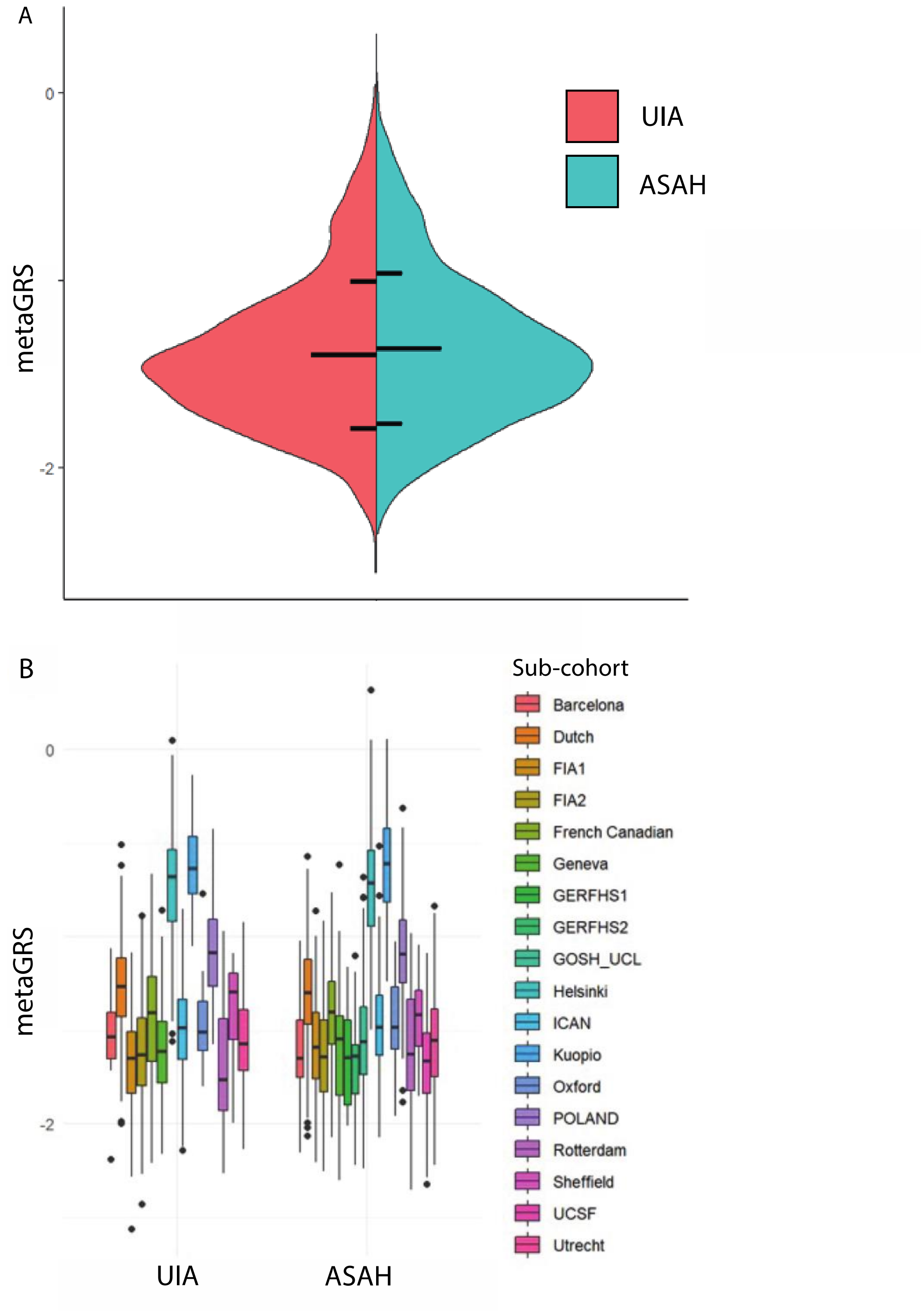

Supplementary Figure 16. Association between metaGRS and size of intracranial aneurysm (IA) at time of aneurysmal subarachnoid hemorrhage (ASAH). A) Darker points are (partially) overlapping data point. Line is the linear regression line of metaGRS on size at ASAH. Shaded area denotes 95% confidence interval. B) Per sub-cohort stratified linear regression of metaGRS on aneurysm size at time of ASAH.

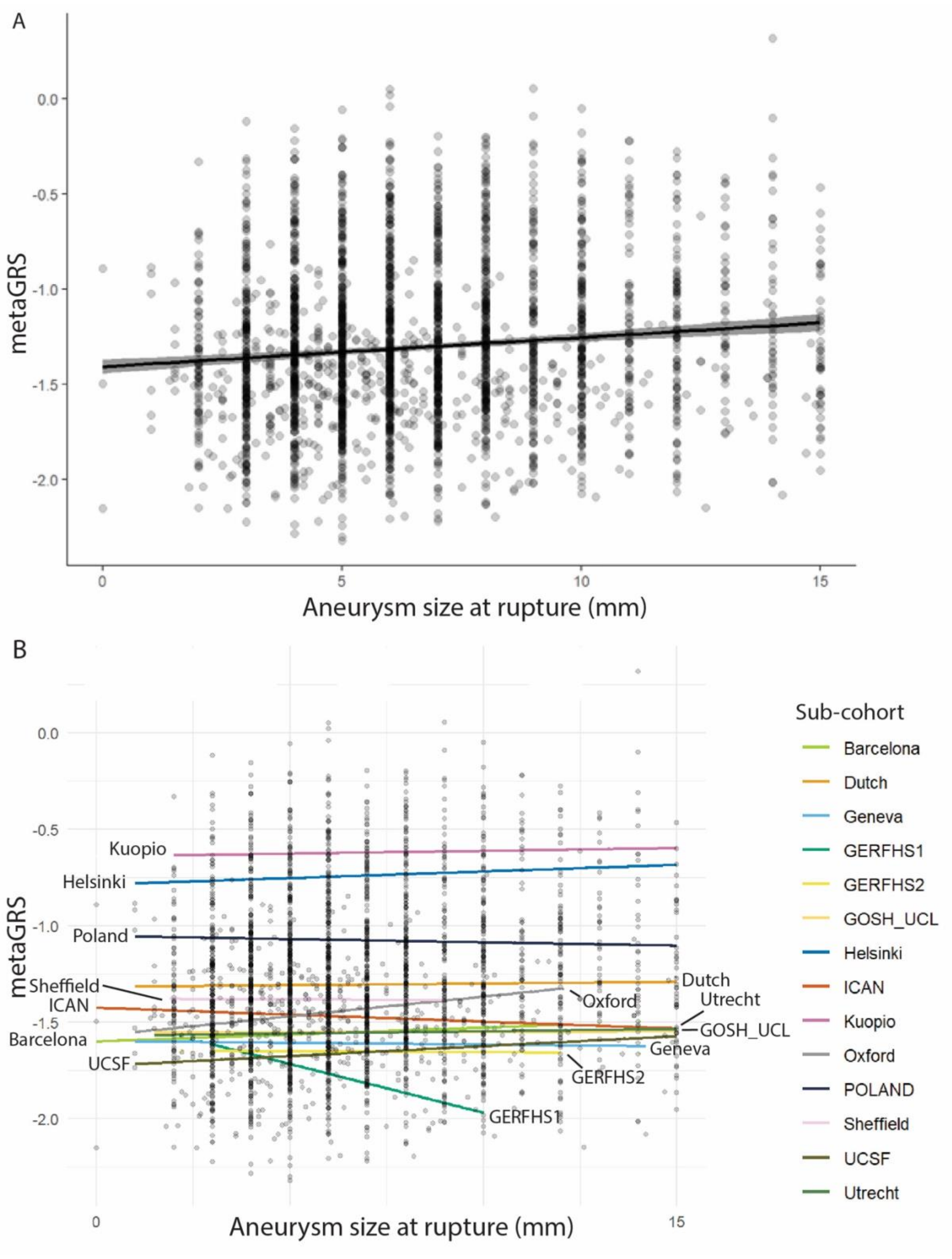

Supplementary Figure 17. Association between metaGRS and hypertension among persons with an intracranial aneurysm (IA, either ruptured or unruptured). A) Violin plot of the distribution of metaGRS in the phenotype cohort stratified by hypertension status. Horizontal lines denote mean and mean ± 1 standard deviation. B) Box plots showing the distribution in each sub-cohort within the phenotype cohort. Boxes contain 25th to 75th percentile and denote the median with a horizontal line. Whiskers denote smallest value greater than 1.5 times the interquartile range below the 25th percentile, and largest value smaller than 1.5 times the interquartile range above the 75th percentile.

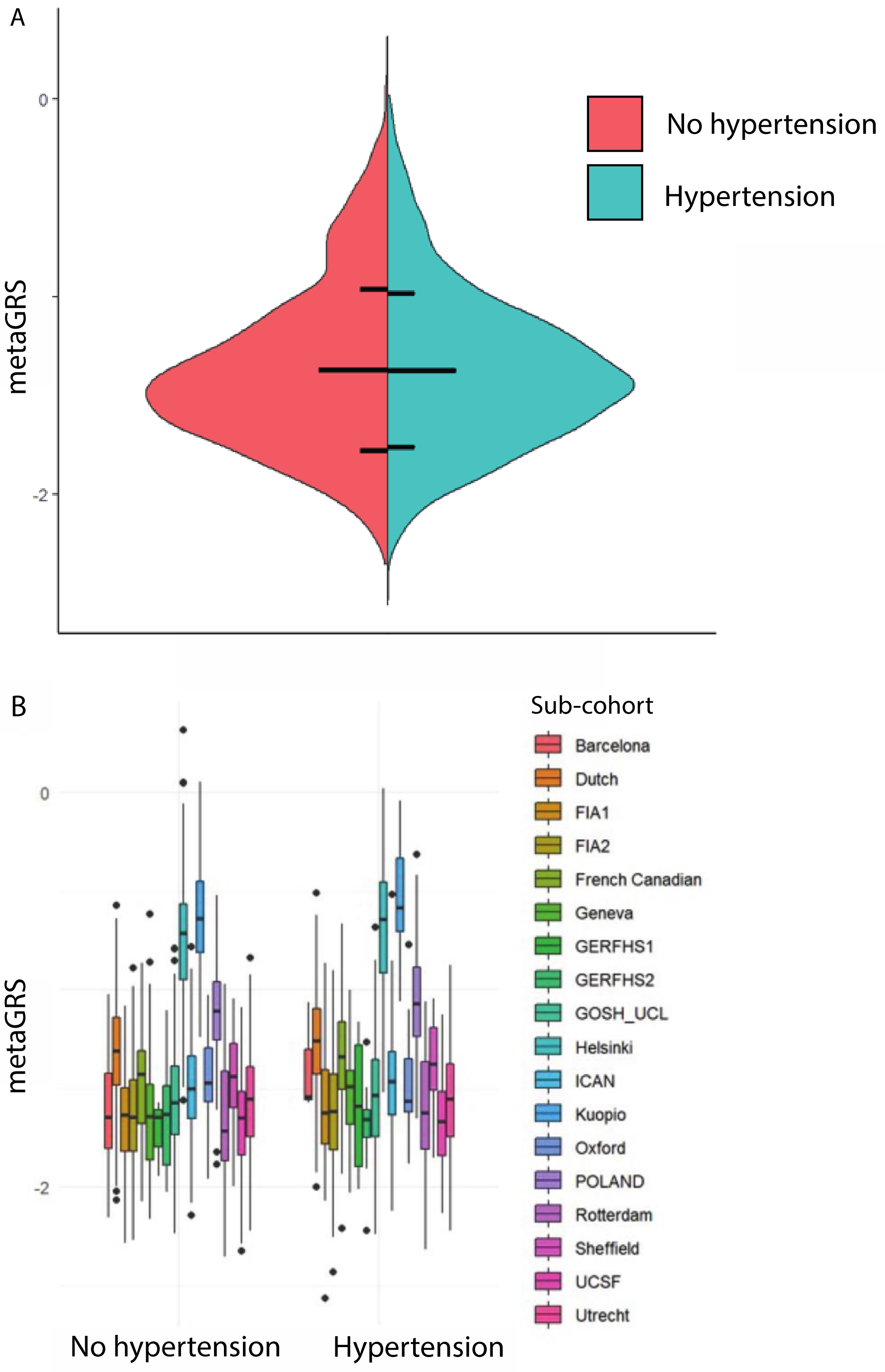

Supplementary Figure 18. Association between metaGRS and smoking among persons with an intracranial aneurysm (IA, either ruptured or unruptured). A) Violin plot of the distribution of metaGRS in the phenotype cohort stratified by smoking history (never, or either a current or past smoker). Horizontal lines denote mean and mean ± 1 standard deviation. B) Box plots showing the distribution in each sub-cohort within the phenotype cohort. Boxes contain 25th to 75th percentile and denote the median with a horizontal line. Whiskers denote smallest value greater than 1.5 times the interquartile range below the 25th percentile, and largest value smaller than 1.5 times the interquartile range above the 75th percentile

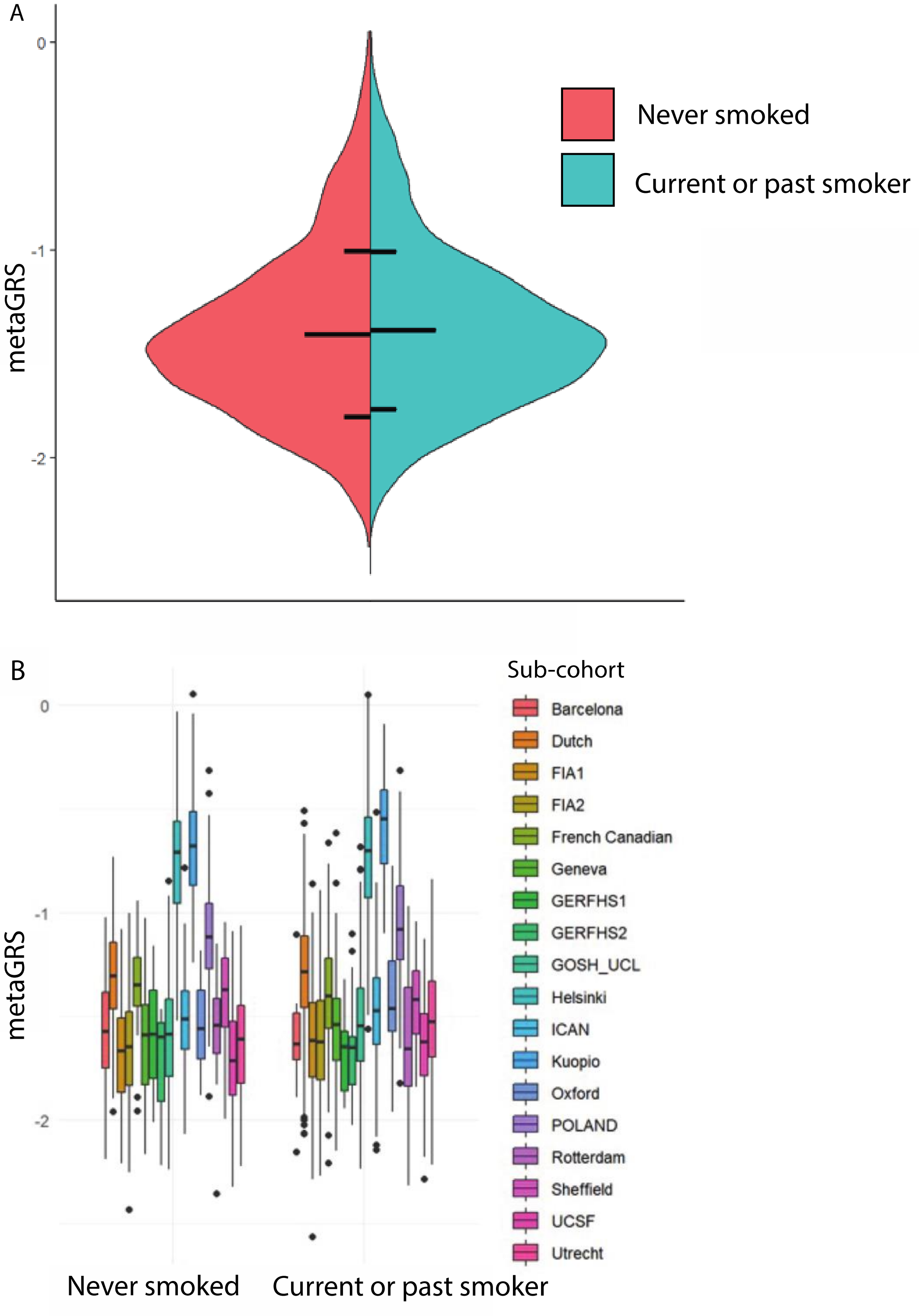

Supplementary Figure 19. Distribution of metaGRS among sub-cohort of the phenotype cohort. Areas are colored according to metaGRS for clarity.

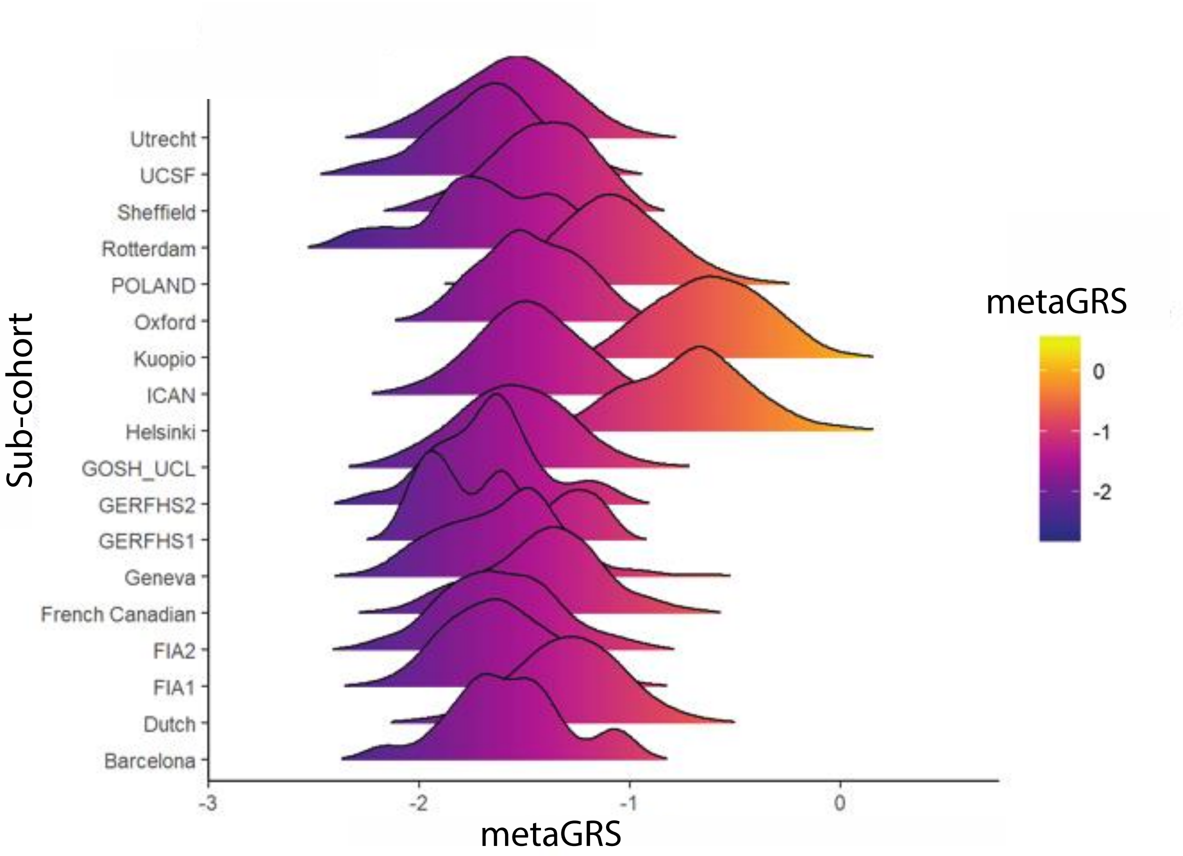

Consortium authors

HUNT All-In Stroke:

Anne Hege Aamodt[1], Anne Heidi Skogholt[2], Ben M Brumpton[2], Cristen J Willer[3], Else C Sandset[4,5], Espen S Kristoffersen[6,7,8], Hanne Ellekjær[9,10], Ingrid Heuch[8], John-Anker Zwart[8,11,2], Jonas B Nielsen[2,3,12], Knut Hagen[9], Kristian Hveem[2,13,14], Lars G Fritsche[15], Laurent F Thomas[2,16,17,18], Linda M Pedersen[8], Maiken E Gabrielsen[2], Oddgeir L Holmen[13], Sigrid Børte[11,2,19], Wei Zhou[20,21]

[1]Department of Neurology, Oslo University Hospital, Oslo, Norway
[2]K. G. Jebsen Center for Genetic Epidemiology, Department of Public Health and Nursing, Faculty of Medicine and Health Sciences, Norwegian University of Science and Technology (NTNU), Trondheim, Norway
[3]Department of Internal Medicine, Division of Cardiovascular Medicine, University of Michigan, Ann Arbor, MI, 48109, USA
[4]Stroke Unit, Department of Neurology, Oslo University Hospital, Oslo, Norway
[5]Research and Development, The Norwegian Air Ambulance Foundation, Norway
[6]Department of General Practice, University of Oslo, Oslo, Norway
[7]Department of Neurology, Akershus University Hospital, Lørenskog, Norway
[8]Department of Research and Innovation, Division of Clinical Neuroscience, Oslo University Hospital, Oslo, Norway
[9]Department of Neuromedicine and Movement Science, Faculty of Medicine and Health Sciences, Norwegian University of Science and Technology (NTNU), Trondheim, Norway
[10]Stroke Unit, Department of Internal Medicine, St. Olavs Hospital, Trondheim University Hospital, Trondheim, Norway
[11]Institute of Clinical Medicine, Faculty of Medicine, University of Oslo, Oslo, Norway
[12]Department of Epidemiology Research, Statens Serum Institut, Copenhagen, Denmark
[13]HUNT Research Center, Department of Public Health and Nursing, Faculty of Medicine and Health Sciences, Norwegian University of Science and Technology (NTNU), Trondheim, Norway
[14]Department of Research, Innovation and Education, St. Olavs Hospital, Trondheim University Hospital, Trondheim, Norway
[15]Center for Statistical Genetics, Department of Biostatistics, University of Michigan, Ann Arbor, MI, 48109, USA
[16]Department of Clinical and Molecular Medicine, Norwegian University of Science and Technology (NTNU), Trondheim, Norway
[17]BioCore - Bioinformatics Core Facility, Norwegian University of Science and Technology (NTNU), Trondheim, Norway
[18]Clinic of Laboratory Medicine, St. Olavs Hospital, Trondheim University Hospital, Trondheim, Norway
[19]Research and Communication Unit for Musculoskeletal Health (FORMI), Department of Research and Innovation, Division of Clinical Neuroscience, Oslo University Hospital, Oslo, Norway
[20]Department of Computational Medicine and Bioinformatics, University of Michigan, Ann Arbor, MI, 48109, USA
[21]Analytic and Translational Genetics Unit, Massachusetts General Hospital, Boston, MA, USA

CADISP Group

Belgium: Departments of Neurology, Erasmus University Hospital, Brussels and Laboratory of Experimental Neurology, ULB, Brussels (Shérine Abboud, Massimo Pandolfo); Department of Neurology, Leuven University Hospial (Vincent Thijs). France: Departments of Neurology, Lille University Hospital-Inserm U1171 (Didier Leys, Marie Bodenant), Sainte-Anne University Hospital, Paris (Fabien Louillet, Emmanuel Touzé, Jean-Louis Mas), Pitié-Salpêtrière University Hospital, Paris (Yves Samson, Sara Leder, Anne Léger, Sandrine Deltour, Sophie Crozier, Isabelle Méresse), Amiens University Hospital (Sandrine Canaple, Olivier Godefroy), Dijon University Hospital (Maurice Giroud, Yannick Béjot), Besançon University Hospital (Pierre Decavel, Elizabeth Medeiros, Paola Montiel, Thierry Moulin, Fabrice Vuillier); Inserm U744, Pasteur Institute, Lille (Jean Dallongeville). Finland : Department of Neurology, Helsinki University Central Hospital, Helsinki (Antti J Metso, Tiina Metso, Turgut Tatlisumak); Germany: Departments of Neurology, Heidelberg University Hospital (Caspar Grond-Ginsbach, Christoph Lichy, Manja Kloss, Inge Werner, Marie-Luise Arnold), University Hospital of Ludwigshafen (Michael Dos Santos, Armin Grau); University Hospital of München (Martin Dichgans); Department of Rehabilitation: Schmieder-Klinik, Heidelberg (Constanze Thomas-Feles, Ralf Weber, Tobias Brandt). Italy: Departments of Neurology: Brescia University Hospital (Alessandro Pezzini, Valeria De Giuli, Filomena Caria, Loris Poli, Alessandro Padovani), Milan University Hospital (Anna Bersano, Silvia Lanfranconi), University of Milano Bicocca, San Gerardo Hospital, Monza, Italy (Simone Beretta, Carlo Ferrarese), Milan Scientific Institute San Raffaele University Hospital (Giacomo Giacolone); Department of Rehabilitation, Santa Lucia Hospital, Rome (Stefano Paolucci). Switzerland: Department of Neurology, Basel University Hospital (Philippe Lyrer, Stefan Engelter, Felix Fluri, Florian Hatz, Dominique Gisler, Leo Bonati, Henrik Gensicke, Margareth Amort). UK: Clinical Neuroscience, St George's University of London (Hugh Markus). USA : Department of Neurology, Salt Lake City, USA (Jennifer Majersik); Department of Neurology, University of Virginia, Charlottesville, USA (Bradford Worrall, Andrew Southerland); Department of Neurology, Baltimore, USA (John Cole, Steven Kittner)

International Consortium for Blood Pressure (ICBP):

Evangelos Evangelou[1,2], Helen R Warren[3,4], He Gao[1,5], Georgios Ntritsos[2], Niki Dimou[2], Tonu Esko[16,17], Reedik Mägi[16], Lili Milani[16], Peter Almgren[18], Thibaud Boutin[19], Stéphanie Debette[20,21], Jun Ding[22], Franco Giulianini[23], Elizabeth G Holliday[24], Anne U Jackson[25], Ruifang Li-Gao[26], Wei-Yu Lin[27], Jian'an Luan[28], Massimo Mangino[29,30], Christopher Oldmeadow[24], Bram Peter Prins[31], Yong Qian[22], Muralidharan Sargurupremraj[21], Nabi Shah[32,33], Praveen Surendran[27], Sébastien Thériault[34,35],

Niek Verweij[17,36,37], Sara M Willems[28], Jing-Hua Zhao[28], Philippe Amouyel[38], John Connell[39], Renée de Mutsert[26], Alex SF Doney[32], Martin Farrall[40,41], Cristina Menni[29], Andrew D Morris[42], Raymond Noordam[43], Guillaume Paré[34], Neil R Poulter[44], Denis C Shields[45], Alice Stanton[46], Simon Thom[47], Gonçalo Abecasis[48], Najaf Amin[49], Dan E Arking[50], Kristin L Ayers[51,52], Caterina M Barbieri[53], Chiara Batini[54], Joshua C Bis[55], Tineka Blake[54], Murielle Bochud[56], Michael Boehnke[25], Eric Boerwinkle[57], Dorret I Boomsma[58], Erwin P Bottinger[59], Peter S Braund[60,61], Marco Brumat[62], Archie Campbell[63,64], Harry Campbell[65], Aravinda Chakravarti[50], John C Chambers[1,5,66-68], Ganesh Chauhan[69], Marina Ciullo[70,71], Massimiliano Cocca[72], Francis Collins[73], Heather J Cordell[51], Gail Davies[74,75], Martin H de Borst[76], Eco J de Geus[58], Ian J Deary[74,75], Joris Deelen[77], Fabiola Del Greco M[78], Cumhur Yusuf Demirkale[79], Marcus Dörr[80,81], Georg B Ehret[50,82], Roberto Elosua[83,84], Stefan Enroth[85], A Mesut Erzurumluoglu[54], Teresa Ferreira[86,87], Mattias Frånberg[88-90], Oscar H Franco[91], Ilaria Gandin[62], Paolo Gasparini[62,72], Vilmantas Giedraitis[92], Christian Gieger[93-95], Giorgia Girotto[62,72], Anuj Goel[40,41], Alan J Gow[74,96], Vilmundur Gudnason[97,98], Xiuqing Guo[99], Ulf Gyllensten[85], Anders Hamsten[88,89], Tamara B Harris[100], Sarah E Harris[63,74], Catharina A Hartman[101], Aki S Havulinna[102,103], Andrew A Hicks[78], Edith Hofer[104,105], Albert Hofman[91,106], Jouke-Jan Hottenga[58], Jennifer E Huffman[19,107,108], Shih-Jen Hwang[107,108], Erik Ingelsson[109,110], Alan James[111,112], Rick Jansen[113], Marjo-Riitta Jarvelin[1,5,114-116], Roby Joehanes[107,117], Åsa Johansson[85], Andrew D Johnson[107,118], Peter K Joshi[65], Pekka Jousilahti[102], J Wouter Jukema[119], Antti Jula[102], Mika Kähönen[120,121], Sekar Kathiresan[17,36,122], Bernard D Keavney[123,124], Kay-Tee Khaw[125], Paul Knekt[102], Joanne Knight[126], Ivana Kolcic[127], Jaspal S Kooner[5,67,68,128], Seppo Koskinen[102], Kati Kristiansson[102], Zoltan Kutalik[56,129], Maris Laan[130], Marty Larson[107], Lenore J Launer[100], Benjamin Lehne[1], Terho Lehtimäki[131,132], David CM Liewald[74,75], Li Lin[82], Lars Lind[133], Cecilia M Lindgren[40,87,134], YongMei Liu[135], Ruth JF Loos[28,59,136], Lorna M Lopez[74,137,138], Yingchang Lu[59], Leo-Pekka Lyytikäinen[131,132], Anubha Mahajan[40], Chrysovalanto Mamasoula[139], Jaume Marrugat[83], Jonathan Marten[19], Yuri Milaneschi[140], Anna Morgan[62], Andrew P Morris[40,141], Alanna C Morrison[142], Peter J Munson[79], Mike A Nalls[143,144], Priyanka Nandakumar[50], Christopher P Nelson[60,61], Teemu Niiranen[102,145], Ilja M Nolte[146], Teresa Nutile[70], Albertine J Oldehinkel[147], Ben A Oostra[49], Paul F O'Reilly[148], Elin Org[16], Sandosh Padmanabhan[64,149], Walter Palmas[150], Aarno Palotie[103,151,152], Alison Pattie[75], Brenda WJH Penninx[140], Markus Perola[102,103,153], Annette Peters[94,95,154], Ozren Polasek[127,155], Peter P Pramstaller[78,156,157], Quang Tri Nguyen[79], Olli T Raitakari[158,159], Rainer Rettig[161], Kenneth Rice[162], Paul M Ridker[23,163], Janina S Ried[94], Harriëtte Riese[147], Samuli Ripatti[103,164], Antonietta Robino[72], Lynda M Rose[23], Jerome I Rotter[99], Igor Rudan[165], Daniela Ruggiero[70,71], Yasaman Saba[166], Cinzia F Sala[53], Veikko Salomaa[102], Nilesh J Samani[60,61], Antti-Pekka Sarin[103], Reinhold Schmidt[104], Helena Schmidt[166], Nick Shrine[54], David Siscovick[167], Albert V Smith[97,98], Harold Snieder[146], Siim Sõber[130], Rossella Sorice[70], John M Starr[74,168], David J Stott[169], David P Strachan[170], Rona J Strawbridge[88,89], Johan Sundström[133], Morris A Swertz[171], Kent D Taylor[99], Alexander Teumer[81,172], Martin D Tobin[54], Maciej Tomaszewski[123,124], Daniela Toniolo[53], Michela Traglia[53], Stella Trompet[119,173], Jaakko Tuomilehto[174-177], Christophe Tzourio[21], André G Uitterlinden[91,178], Ahmad Vaez[146,179], Peter J van der Most[146], Cornelia M van Duijn[49], Germaine C Verwoert[91], Veronique Vitart[19], Uwe Völker[81,180], Peter Vollenweider[181], Dragana Vuckovic[62,182], Hugh Watkins[40,41], Sarah H Wild[183], Gonneke Willemsen[58], James F Wilson[19,65], Alan F Wright[19], Jie Yao[99], Tatijana Zemunik[184], Weihua Zhang[1,67], John R Attia[24], Adam S Butterworth[27,185], Daniel I Chasman[23,163], David Conen[186,187], Francesco Cucca[188,189], John Danesh[27,185], Caroline Hayward[19], Joanna MM Howson[27], Markku Laakso[190], Edward G Lakatta[191], Claudia Langenberg[28], Olle Melander[18], Dennis O Mook-Kanamori[26,192], Colin NA Palmer[32], Lorenz Risch[193-195], Robert A Scott[28], Rodney J Scott[24], Peter Sever[128], Tim D Spector[29], Pim van der Harst[196], Nicholas J Wareham[28], Eleftheria Zeggini[31], Daniel Levy[107,118], Patricia B Munroe[3,4], Christopher Newton-Cheh[134,197,198], Morris J Brown[3,4], Andres Metspalu[16], Bruce M. Psaty[201,202], Louise V Wain[54], Paul Elliott[1,5,203-205], Mark J Caulfield[3,4]

1. Department of Epidemiology and Biostatistics, Imperial College London, London, UK.
2. Department of Hygiene and Epidemiology, University of Ioannina Medical School, Ioannina, Greece.
3. William Harvey Research Institute, Barts and The London School of Medicine and Dentistry, Queen Mary University of London, London, UK.
4. National Institute for Health Research, Barts Cardiovascular Biomedical Research Center, Queen Mary University of London, London, UK.
5. MRC-PHE Centre for Environment and Health, Imperial College London, London, UK.
7. Division of Epidemiology, Department of Medicine, Institute for Medicine and Public Health, Vanderbilt Genetics Institute, Vanderbilt University Medical Center, Tennessee Valley Healthcare System (626)/Vanderbilt University, Nashville, TN, USA.
8. Vanderbilt Genetics Institute, Vanderbilt Epidemiology Center, Department of Obstetrics and Gynecology, Vanderbilt University Medical Center; Tennessee Valley Health Systems VA, Nashville, TN, USA.
9. Department of Epidemiology, Emory University Rollins School of Public Health, Atlanta, GA, USA.
10. Department of Biomedical Informatics, Emory University School of Medicine, Atlanta, GA, USA.
11. Massachusetts Veterans Epidemiology Research and Information Center (MAVERIC), VA Boston Healthcare System, Boston, USA.
12. Division of Aging, Department of Medicine, Brigham and Women’s Hospital, Boston, MA, Department of Medicine, Harvard Medical School, Boston, MA, USA.
13. Atlanta VAMC and Emory Clinical Cardiovascular Research Institute, Atlanta, GA, USA.
14. VA Palo Alto Health Care System; Division of Cardiovascular Medicine, Stanford University School of Medicine, CA, USA.
15. Nephrology Section, Memphis VA Medical Center and University of Tennessee Health Science Center, Memphis, TN, USA.

16. Estonian Genome Center, University of Tartu, Tartu, Estonia.
17. Program in Medical and Population Genetics, Broad Institute of Harvard and MIT, Cambridge, MA, USA.
18. Department Clinical Sciences, Malmö, Lund University, Malmö, Sweden.
19. MRC Human Genetics Unit, MRC Institute of Genetics and Molecular Medicine, University of Edinburgh, Western General Hospital, Edinburgh, Scotland, UK
20. Department of Neurology, Bordeaux University Hospital, Bordeaux, France.
21. Univ. Bordeaux, Inserm, Bordeaux Population Health Research Center, CHU Bordeaux, Bordeaux, France.
22. Laboratory of Genetics and Genomics, NIA/NIH , Baltimore, MD, USA.
23. Division of Preventive Medicine, Brigham and Women's Hospital, Boston, MA, USA.
24. Hunter Medical Reseach Institute and Faculty of Health, University of Newcastle, New Lambton Heights, New South Wales, Australia.
25. Department of Biostatistics and Center for Statistical Genetics, University of Michigan, Ann Arbor, MI, USA.
26. Department of Clinical Epidemiology, Leiden University Medical Center, Leiden, the Netherlands.
27. MRC/BHF Cardiovascular Epidemiology Unit, Department of Public Health and Primary Care, University of Cambridge, Cambridge, UK.
28. MRC Epidemiology Unit, University of Cambridge School of Clinical Medicine, Cambridge, UK.
29. Department of Twin Research and Genetic Epidemiology, Kings College London, London, UK.
30. NIHR Biomedical Research Centre at Guy’s and St Thomas’ Foundation Trust, London, UK.
31. Wellcome Trust Sanger Institute, Hinxton, UK.
32. Division of Molecular and Clinical Medicine, School of Medicine, University of Dundee, UK.
33. Department of Pharmacy, COMSATS Institute of Information Technology, Abbottabad, Pakistan.
34. Department of Pathology and Molecular Medicine, McMaster University, Hamilton, Canada.
35. Institut universitaire de cardiologie et de pneumologie de Québec-Université Laval, , Quebec City, Canada.
36. Cardiovascular Research Center and Center for Human Genetic Research, Massachusetts General Hospital, Boston, Massachusetts, MA, USA.
37. University of Groningen, University Medical Center Groningen, Department of Cardiology, Groningen, The Netherlands.
38. University of Lille, Inserm, Centre Hosp. Univ Lille, Institut Pasteur de Lille, UMR1167 - RID-AGE - Risk factors and molecular determinants of aging-related diseases, Epidemiology and Public Health Department, Lille, France.
39. University of Dundee, Ninewells Hospital & Medical School, Dundee, , UK.
40. Wellcome Trust Centre for Human Genetics, University of Oxford, Oxford, UK.
41. Division of Cardiovascular Medicine, Radcliffe Department of Medicine, University of Oxford, Oxford, UK.
42. Usher Institute of Population Health Sciences and Informatics, University of Edinburgh, UK.
43. Department of Internal Medicine, Section Gerontology and Geriatrics, Leiden University Medical Center, Leiden, The Netherlands.
44. Imperial Clinical Trials Unit, Stadium House, 68 Wood Lane, London, UK.
45. School of Medicine, University College Dublin, Ireland.
46. Molecular and Cellular Therapeutics, Royal College of Surgeons in Ireland, Dublin, Ireland.
47. International Centre for Circulatory Health, Imperial College London, London, UK.
48. Center for Statistical Genetics, Dept. of Biostatistics, SPH II, Washington Heights, Ann Arbor, MI, USA.
49. Genetic Epidemiology Unit, Department of Epidemiology, Erasmus MC, Rotterdam, the Netherlands.
50. Center for Complex Disease Genomics, McKusick-Nathans Institute of Genetic Medicine, Johns Hopkins University School of Medicine, Baltimore, MD, USA.
51. Institute of Genetic Medicine, Newcastle University, Newcastle upon Tyne, UK.
52. Sema4, a Mount Sinai venture, Stamford, CT, USA.
53. Division of Genetics and Cell Biology, San Raffaele Scientific Institute, Milano, Italy.
54. Department of Health Sciences, University of Leicester, Leicester, UK.
55. Cardiovascular Health Research Unit, Department of Medicine, University of Washington, Seattle, WA, USA.
56. Institute of Social and Preventive Medicine, University Hospital of Lausanne, Lausanne, Switzerland.
57. Human Genetics Center, School of Public Health, The University of Texas Health Science Center at Houston and Human Genome Sequencing Center, Baylor College of Medicine, One Baylor Plaza, Houston, TX, USA.
58. Department of Biological Psychology, Vrije Universiteit Amsterdam, EMGO+ institute, VU University medical center, Amsterdam, the Netherlands.
59. The Charles Bronfman Institute for Personalized Medicine, Icachn School of Medicine at Mount Sinai, NY, USA.
60. Department of Cardiovascular Sciences, University of Leicester, Leicester, UK.
61. NIHR Leicester Biomedical Research Centre, Glenfield Hospital, Groby Road, Leicester, UK.
62. Department of Medical, Surgical and Health Sciences, University of Trieste, , Trieste, Italy.
63. Medical Genetics Section, Centre for Genomic and Experimental Medicine, Institute of Genetics and Molecular Medicine, University of Edinburgh, Edinburgh, UK.
64. Generation Scotland, Centre for Genomic and Experimental Medicine, University of Edinburgh, Edinburgh, UK.
65. Centre for Global Health Research, Usher Institute of Population Health Sciences and Informatics, University of Edinburgh, Edinburgh, Scotland, UK
66. Lee Kong Chian School of Medicine, Nanyang Technological University, Singapore, Singapore.

67. Department of Cardiology, Ealing Hospital, Middlesex, UK.
68. Imperial College Healthcare NHS Trust, London, UK.
69. Centre for Brain Research, Indian Institute of Science, Bangalore, India.
70. Institute of Genetics and Biophysics "A. Buzzati-Traverso", CNR, Napoli, Italy.
71. IRCCS Neuromed, Pozzilli, Isernia, Italy.
72. Institute for Maternal and Child Health IRCCS Burlo Garofolo, Trieste, Italy.
73. Medical Genomics and Metabolic Genetics Branch, National Human Genome Research Institute, NIH, Bethesda, MD, USA.
74. Centre for Cognitive Ageing and Cognitive Epidemiology, University of Edinburgh, 7 George Square, Edinburgh, UK.
75. Department of Psychology, University of Edinburgh, 7 George Square, Edinburgh, UK.
76. Department of Internal Medicine, Division of Nephrology, University of Groningen, University Medical Center Groningen, Groningen, The Netherlands.
77. Department of Molecular Epidemiology, Leiden University Medical Center, Leiden, the Netherlands.
78. Institute for Biomedicine, Eurac Research, Bolzano, Italy - Affiliated Institute of the University of Lübeck, Lübeck, Germany.
79. Mathematical and Statistical Computing Laboratory, Office of Intramural Research, Center for Information Technology, National Institutes of Health, Bethesda, MD, USA.
80. Department of Internal Medicine B, University Medicine Greifswald, Greifswald, Germany.
81. DZHK (German Centre for Cardiovascular Research), partner site Greifswald, Greifswald, Germany.
82. Cardiology, Department of Medicine, Geneva University Hospital, Geneva, Switzerland.
83. CIBERCV & Cardiovascular Epidemiology and Genetics, IMIM. Dr Aiguader 88, Barcelona, Spain.
84. Faculty of Medicine, Universitat de Vic-Central de Catalunya, Vic, Spain.
85. Department of Immunology, Genetics and Pathology, Uppsala Universitet, Science for Life Laboratory, Uppsala, Sweden.
86. Wellcome Centre for Human Genetics, University of Oxford, Roosevelt Drive, Oxford, UK.
87. Big Data Institute, Li Ka Shing Center for Health for Health Information and Discovery, Oxford University, Old Road, Oxford, UK.
88. Cardiovascular Medicine Unit, Department of Medicine Solna, Karolinska Institutet, Stockholm, Sweden.
89. Centre for Molecular Medicine, L8:03, Karolinska Universitetsjukhuset, Solna, Sweden.
90. Department of Numerical Analysis and Computer Science, Stockholm University, Stockholm, Sweden.
91. Department of Epidemiology, Erasmus MC, Rotterdam, the Netherlands.
92. Department of Public Health and Caring Sciences, Geriatrics, Uppsala, Sweden.
93. Research Unit of Molecular Epidemiology, Helmholtz Zentrum München, German Research Center for Environmental Health, Neuherberg, Germany.
94. Institute of Epidemiology, Helmholtz Zentrum München, German Research Center for Environmental Health, Neuherberg, Germany.
95. German Center for Diabetes Research (DZD e.V.), Neuherberg, Germany.
96. Department of Psychology, School of Social Sciences, Heriot-Watt University, Edinburgh, UK.
97. Faculty of Medicine, University of Iceland, Reykjavik, Iceland.
98. Icelandic Heart Association, Kopavogur, Iceland.
99. The Institute for Translational Genomics and Population Sciences, Department of Pediatrics, LABioMed at Harbor-UCLA Medical Center, Torrance, CA, USA.
100. Intramural Research Program, Laboratory of Epidemiology, Demography, and Biometry, National Institute on Aging, Bethesda, MD, USA.
101. Department of Psychiatry, University of Groningen, University Medical Center Groningen, Groningen, The Netherlands.
102. Department of Public Health Solutions, National Institute for Health and Welfare (THL), Helsinki, Finland.
103. Institute for Molecular Medicine Finland (FIMM), University of Helsinki, Helsinki, Finland.
104. Clinical Division of Neurogeriatrics, Department of Neurology, Medical University of Graz, Graz, Austria.
105. Institute for Medical Informatics, Statistics and Documentation, Medical University of Graz, Graz, Austria.
106. Department of Epidemiology, Harvard T.H. Chan School of Public Health, Boston, MA, USA.
107. National Heart, Lung and Blood Institute's Framingham Heart Study, Framingham, MA, USA.
108. The Population Science Branch, Division of Intramural Research, National Heart Lung and Blood Institute national Institute of Health, Bethesda, MD, USA.
109. Department of Medical Sciences, Molecular Epidemiology and Science for Life Laboratory, Uppsala University, Uppsala, Sweden.
110. Division of Cardiovascular Medicine, Department of Medicine, Stanford University School of Medicine, Stanford, CA USA.
111. Department of Pulmonary Physiology and Sleep, Sir Charles Gairdner Hospital, Hospital Avenue, Nedlands, Australia.
112. School of Medicine and Pharmacology, University of Western Australia.

113. Department of Psychiatry, VU University Medical Center, Amsterdam Neuroscience, Amsterdam, the Netherlands.
114. Biocenter Oulu, University of Oulu, Oulu, Finland.
115. Center For Life-course Health Research, University of Oulu, Oulu Finland.
116. Unit of Primary Care, Oulu University Hospital, Oulu, Oulu, Finland.
117. Hebrew SeniorLife, Harvard Medical School, Boston, MA, USA.
118. Population Sciences Branch, National Heart, Lung and Blood Institute, National Institutes of Health, Bethesda, MD, USA.
119. Department of Cardiology, Leiden University Medical Center, Leiden, the Netherlands.
120. Department of Clinical Physiology, Tampere University Hospital, Tampere, Finland.
121. Department of Clinical Physiology, Finnish Cardiovascular Research Center - Tampere, Faculty of Medicine and Life Sciences, University of Tampere, Tampere, Finland.
122. Broad Institute of the Massachusetts Institute of Technology and Harvard University, Cambridge, MA, USA.
123. Division of Cardiovascular Sciences, Faculty of Biology, Medicine and Health, The University of Manchester, Manchester, UK.
124. Division of Medicine, Manchester University NHS Foundation Trust, Manchester Academic Health Science Centre, Manchester, UK
125. Department of Public Health and Primary Care, Institute of Public Health, University of Cambridge, Cambridge, UK.
126. Data Science Institute and Lancaster Medical School, Lancaster, UK.
127. Department of Public Health, Faculty of Medicine, University of Split, Croatia.
128. National Heart and Lung Institute, Imperial College London, London, UK.
129. Swiss Institute of Bioinformatics, Lausanne, Switzerland.
130. Institute of Biomedicine and Translational Medicine, University of Tartu, Tartu, Estonia.
131. Department of Clinical Chemistry, Fimlab Laboratories, Tampere, Finland.
132. Department of Clinical Chemistry, Finnish Cardiovascular Research Center - Tampere, Faculty of Medicine and Life Sciences, University of Tampere, Tampere, Finland
133. Department of Medical Sciences, Cardiovascular Epidemiology, Uppsala University, Uppsala, Sweden.
134. Program in Medical and Population Genetics, Broad Institute, Cambridge, MA, USA.
135. Division of Public Health Sciences, Wake Forest School of Medicine, Winston-Salem, NC, USA.
136. Mindich Child health Development Institute, The Icahn School of Medicine at Mount Sinai, New York, NY, USA.
137. Department of Psychiatry, Royal College of Surgeons in Ireland, Education and Research Centre, Beaumont Hospital, Dublin, Ireland.
138. University College Dublin, UCD Conway Institute, Centre for Proteome Research, UCD, Belfield, Dublin, Ireland.
139. Institute of Health and Society, Newcastle University, Newcastle upon Tyne, UK.
140. Department of Psychiatry, Amsterdam Public Health and Amsterdam Neuroscience, VU University Medical Center/GGZ inGeest, Amsterdam, The Netherlands.
141. Department of Biostatistics, University of Liverpool, Block F, Waterhouse Building, Liverpool, UK.
142. Department of Epidemiology, Human Genetics and Environmental Sciences, School of Public Health, University of Texas Health Science Center at Houston, Houston, TX, USA.
143. Data Tecnica International, Glen Echo, MD, USA.
144. Laboratory of Neurogenetics, National Institute on Aging, Bethesda, USA.
145. Department of Medicine, Turku University Hospital and University of Turku, Finland.
146. Department of Epidemiology, University of Groningen, University Medical Center Groningen, Groningen, The Netherlands.
147. Interdisciplinary Center Psychopathology and Emotion regulation (ICPE), University of Groningen, University Medical Center Groningen, Groningen, The Netherlands.
148. SGDP Centre, Institute of Psychiatry, Psychology and Neuroscience, King's College London, London, UK.
149. British Heart Foundation Glasgow Cardiovascular Research Centre, Institute of Cardiovascular and Medical Sciences, College of Medical, Veterinary and Life Sciences, University of Glasgow, Glasgow, UK.
150. Department of Medicine, Columbia University Medical Center, New York, NY, USA.
151. Analytic and Translational Genetics Unit, Department of Medicine, Department of Neurology and Department of Psychiatry Massachusetts General Hospital, Boston, MA, USA.
152. The Stanley Center for Psychiatric Research and Program in Medical and Population Genetics, The Broad Institute of MIT and Harvard, Cambridge, MA, USA.
153. University of Tartu, Tartu, Estonia.
154. German Center for Cardiovascular Disease Research (DZHK), partner site Munich, Neuherberg, Germany.
155. Psychiatric hospital “Sveti Ivan”, Zagreb, Croatia.
156. Department of Neurology, General Central Hospital, Bolzano, Italy.
157. Department of Neurology, University of Lübeck, Lübeck, Germany.
158. Department of Clinical Physiology and Nuclear Medicine, Turku University Hospital, Turku, Finland.
159. Research Centre of Applied and Preventive Cardiovascular Medicine, University of Turku, Turku, Finland.
161. Institute of Physiology, University Medicine Greifswald, Karlsburg, Germany.

162. Department of Biostatistics University of Washington, Seattle, WA, USA.
163. Harvard Medical School, Boston MA.
164. Public health, Faculty of Medicine, University of Helsinki, Finland
165. Centre for Global Health Research, Usher Institute of Population Health Sciences and Informatics, University of Edinburgh, Scotland, UK.
166. Gottfried Schatz Research Center for Cell Signaling, Metabolism & Aging, Molecular Biology and Biochemistry, Medical University of Graz, Graz, Austria.
167. The New York Academy of Medicine, New York, NY, USA.
168. Alzheimer Scotland Dementia Research Centre, University of Edinburgh, Edinburgh, UK.
169. Institute of Cardiovascular and Medical Sciences, Faculty of Medicine, University of Glasgow, United Kingdom.
170. Population Health Research Institute, St George's, University of London, London, UK.
171. Department of Genetics, University of Groningen, University Medical Center Groningen, Groningen, The Netherlands.
172. Institute for Community Medicine, University Medicine Greifswald, Greifswald, Germany.
173. Department of Gerontology and Geriatrics, Leiden University Medical Center, Leiden, the Netherlands.
174. Dasman Diabetes Institute, Dasman, Kuwait.
175. Chronic Disease Prevention Unit, National Institute for Health and Welfare, Helsinki, Finland.
176. Department of Public Health, University of Helsinki, Helsinki, Finland.
177. Saudi Diabetes Research Group, King Abdulaziz University, Jeddah, Saudi Arabia.
178. Department of Internal Medicine, Erasmus MC, Rotterdam, the Netherlands.
179. Research Institute for Primordial Prevention of Non-communicable Disease, Isfahan University of Medical Sciences, Isfahan, Iran.
180. Interfaculty Institute for Genetics and Functional Genomics, University Medicine Greifswald, Greifswald, Germany.
181. Department of Internal Medicine, University Hospital, CHUV, Lausanne, Switzerland.
182. Experimental Genetics Division, Sidra Medical and Research Center, Doha, Qatar.
183. Centre for Population Health Sciences, Usher Institute of Population Health Sciences and Informatics, University of Edinburgh, Scotland, UK
184. Department of Biology, Faculty of Medicine, University of Split, Croatia.
185. The National Institute for Health Research Blood and Transplant Research Unit in Donor Health and Genomics, University of Cambridge, UK.
186. Division of Cardiology, University Hospital, Basel, Switzerland.
187. Division of Cardiology, Department of Medicine, McMaster University, Hamilton, Canada.
188. Institute of Genetic and Biomedical Research, National Research Council (CNR), Monserrato, Cagliari, Italy.
189. Department of Biomedical Sciences, University of Sassari, Sassari, Italy.
190. Institute of Clinical Medicine, Internal Medicine, University of Eastern Finland and Kuopio University Hospital, Kuopio, Finland.
191. Laboratory of Cardiovascular Science, NIA/NIH , Baltimore, MD, USA.
192. Department of Public Health and Primary Care, Leiden University Medical Center, Leiden, the Netherlands.
193. Labormedizinisches Zentrum Dr. Risch, Schaan, Liechtenstein.
194. Private University of the Principality of Liechtenstin, Triesen, Liechtenstein.
195. University Insitute of Clinical Chemistry, Inselspital, Bern University Hospital, University of Bern, Bern, Switzerland.
196. Department of Cardiology, University of Groningen, University Medical Center Groningen, Groningen, The Netherlands.
197. Center for Genomic Medicine, Massachusetts General Hospital, Boston, MA, USA.
198. Cardiovascular Research Center, Massachusetts General Hospital, Boston, MA, USA.
201. Cardiovascular Health Research Unit, Departments of Medicine, Epidemiology and Health Services, University of Washington, Seattle, WA, USA.
202. Kaiser Permanente Washington Health Research Institute, Seattle, WA, USA.
203. National Institute for Health Research Imperial Biomedical Research Centre, Imperial College Healthcare NHS Trust and Imperial College London, London, UK.
204. UK Dementia Research Institute (UK DRI) at Imperial College London, London, UK
205. Health Data Research-UK London substantive site, London, U.K

International Headache Genetic Consortium (IHGC):

Padhraig Gormley[1–4,81], Verneri Anttila[2,3,5,81], Bendik S Winsvold[6–8], Priit Palta[9], Tonu Esko[2,10,11], Tune H Pers[2,11–13], Kai-How Farh[2,5,14], Ester Cuenca-Leon[1–3,15], Mikko Muona[9,16–18], Nicholas A Furlotte[19], Tobias Kurth[20,21], Andres Ingason[22], George McMahon[23], Lannie Ligthart[24], Gisela M Terwindt[25], Mikko Kallela[26], Tobias M Freilinger[27,28], Caroline Ran[29], Scott G Gordon[30], Anine H Stam[25], Stacy Steinberg[22], Guntram Borck[31], Markku Koiranen[32], Lydia Quaye[33], Hieab H H Adams[34,35], Terho Lehtimäki[36], Antti-Pekka Sarin[9], Juho Wedenoja[37], David A Hinds[19], Julie E Buring[21,38], Markus Schürks[39], Paul M Ridker[21,38], Maria Gudlaug Hrafnsdottir[40], Hreinn Stefansson[22], Susan M Ring[23], Jouke-Jan Hottenga[24], Brenda W J H Penninx[41], Markus Färkkilä[26], Ville Artto[26], Mari Kaunisto[9], Salli Vepsäläinen[26], Rainer Malik[28], Andrew C Heath[42], Pamela A

F Madden[42], Nicholas G Martin[30], Grant W Montgomery[30], Mitja I Kurki[1–3,9,43], Mart Kals[10], Reedik Mägi[10], Kalle Pärn[10], Eija Hämäläinen[9], Hailiang Huang[2,3,5], Andrea E Byrnes[2,3,5], Lude Franke[44], Jie Huang[4], Evie Stergiakouli[23], Phil H Lee[1–3], Cynthia Sandor[45], Caleb Webber[45], Zameel Cader[46,47], Bertram Muller-Myhsok[48,76,93], Stefan Schreiber[49], Thomas Meitinger[50,51], Johan G Eriksson[52,53], Veikko Salomaa[53], Kauko Heikkilä[54], Elizabeth Loehrer[34,55], Andre G Uitterlinden[56], Albert Hofman[34], Cornelia M van Duijn[34], Lynn Cherkas[33], Linda M Pedersen[6], Audun Stubhaug[57,58], Christopher S Nielsen[57,59], Minna Männikkö[32], Evelin Mihailov[10], Lili Milani[10], Hartmut Göbel[60], Ann-Louise Esserlind[61], Anne Francke Christensen[61], Thomas Folkmann Hansen[62], Thomas Werge[63–65], International Headache Genetics Consortium[66], Jaakko Kaprio[9,37,67], Arpo J Aromaa[53], Olli Raitakari[68,69], M Arfan Ikram[34,35,70], Tim Spector[33], Marjo-Riitta Järvelin[32,71–73], Andres Metspalu[10], Christian Kubisch[74], David P Strachan[75], Michel D Ferrari[25], Andrea C Belin[29], Martin Dichgans[28,76], Maija Wessman[9,16], Arn M J M van den Maagdenberg[25,77], John-Anker Zwart[6–8], Dorret I Boomsma[24], George Davey Smith[23], Kari Stefansson[22,78], Nicholas Eriksson[19], Mark J Daly[2,3,5], Benjamin M Neale[2,3,5,82], Jes Olesen[61,82], Daniel I Chasman[21,38,82], Dale R Nyholt[79,82] & Aarno Palotie[1–5,9,80,82]

[1]Psychiatric and Neurodevelopmental Genetics Unit, Massachusetts General Hospital and Harvard Medical School, Boston, Massachusetts, USA. [2]Medical and Population Genetics Program, Broad Institute of MIT and Harvard, Cambridge, Massachusetts, USA. [3]Stanley Center for Psychiatric Research, Broad Institute of MIT and Harvard, Cambridge, Massachusetts, USA. [4]Wellcome Trust Sanger Institute, Wellcome Trust Genome Campus, Hinxton, UK. [5]Analytic and Translational Genetics Unit, Massachusetts General Hospital and Harvard Medical School, Boston, Massachusetts, USA. [6]FORMI, Oslo University Hospital, Oslo, Norway. [7]Department of Neurology, Oslo University Hospital, Oslo, Norway. [8]Institute of Clinical Medicine, University of Oslo, Oslo, Norway. [9]Institute for Molecular Medicine Finland (FIMM), University of Helsinki, Helsinki, Finland. [10]Estonian Genome Center, University of Tartu, Tartu, Estonia. [11]Division of Endocrinology, Boston Children's Hospital, Boston, Massachusetts, USA. [12]Department of Epidemiology Research, Statens Serum Institut, Copenhagen, Denmark. [13]Novo Nordisk Foundation Center for Basic Metabolic Research, University of Copenhagen, Copenhagen, Denmark. [14]Illumina, San Diego, California, USA. [15]Pediatric Neurology, Vall d'Hebron Research Institute, Barcelona, Spain. [16]Folkhälsan Institute of Genetics, Helsinki, Finland. [17]Neuroscience Center, University of Helsinki, Helsinki, Finland. [18]Molecular Neurology Research Program, Research Programs Unit, University of Helsinki, Helsinki, Finland. [19]23andMe, Inc., Mountain View, California, USA. [20]Institute of Public Health, Charité–Universitätsmedizin Berlin, Berlin, Germany. [21]Division of Preventive Medicine, Brigham and Women's Hospital, Boston, Massachusetts, USA. [22]deCODE Genetics, Reykjavik, Iceland. [23]Medical Research Council (MRC) Integrative Epidemiology Unit, University of Bristol, Bristol, UK. [24]Department of Biological Psychology, Vrije Universiteit, Amsterdam, the Netherlands. [25]Department of Neurology, Leiden University Medical Centre, Leiden, the Netherlands. [26]Department of Neurology, Helsinki University Central Hospital, Helsinki, Finland. [27]Department of Neurology and Epileptology, Hertie-Institute for Clinical Brain Research, University of Tuebingen, Tuebingen, Germany. [28]Institute for Stroke and Dementia Research, Klinikum der Universität München, Ludwig-Maximilians-Universität München, Munich, Germany. [29]Department of Neuroscience, Karolinska Institutet, Stockholm, Sweden. [30]Department of Genetics and Computational Biology, QIMR Berghofer Medical Research Institute, Brisbane, Queensland, Australia. [31]Institute of Human Genetics, Ulm University, Ulm, Germany. [32]Center for Life Course Epidemiology and Systems Medicine, University of Oulu, Oulu, Finland. [33]Department of Twin Research and Genetic Epidemiology, King's College London, London, UK. [34]Department of Epidemiology, Erasmus University Medical Center, Rotterdam, the Netherlands. [35]Department of Radiology, Erasmus University Medical Center, Rotterdam, the Netherlands. [36]Department of Clinical Chemistry, Fimlab Laboratories, School of Medicine, University of Tampere, Tampere, Finland. [37]Department of Public Health, University of Helsinki, Helsinki, Finland. [38]Harvard Medical School, Boston, Massachusetts, USA. [39]Department of Neurology, University Duisburg–Essen, Essen, Germany. [40]Landspitali University Hospital, Reykjavik, Iceland. [41]Department of Psychiatry, VU University Medical Centre, Amsterdam, the Netherlands. [42]Department of Psychiatry, Washington University School of Medicine, St. Louis, Missouri, USA. [43]Department of Neurosurgery, NeuroCenter, Kuopio University Hospital, Kuopio, Finland. [44]Department of Genetics, University Medical Center Groningen, University of Groningen, Groningen, the Netherlands. [45]MRC Functional Genomics Unit, Department of Physiology, Anatomy & Genetics, Oxford University, Oxford, UK. [46]Nuffield Department of Clinical Neuroscience, University of Oxford, Oxford, UK. [47]Oxford Headache Centre, John Radcliffe Hospital, Oxford, UK. [48]Max Planck Institute of Psychiatry, Munich, Germany. [49]Institute of Clinical Molecular Biology, Christian Albrechts University, Kiel, Germany. [50]Institute of Human Genetics, Helmholtz Zentrum München, Neuherberg, Germany. [51]Institute of Human Genetics, Technische Universität München, Munich, Germany. [52]Department of General Practice and Primary Health Care, University of Helsinki and Helsinki University Hospital, Helsinki, Finland. [53]National Institute for Health and Welfare, Helsinki, Finland. [54]Institute of Clinical Medicine, University of Helsinki, Helsinki, Finland. [55]Department of Environmental Health, Harvard T.H. Chan School of Public Health, Boston, Massachusetts, USA. [56]Department of Internal Medicine, Erasmus University Medical Center, Rotterdam, the Netherlands. [57]Department of

Pain Management and Research, Oslo University Hospital, Oslo, Norway. [58]Medical Faculty, University of Oslo, Oslo, Norway. [59]Department of Ageing and Health, Norwegian Institute of Public Health, Oslo, Norway. [60]Kiel Pain and Headache Center, Kiel, Germany. [61]Danish Headache Center, Department of Neurology, Rigshospitalet, Glostrup Hospital, University of Copenhagen, Copenhagen, Denmark. [62]Institute of Biological Psychiatry, Mental Health Center Sct. Hans, University of Copenhagen, Roskilde, Denmark. [63]Institute of Biological Psychiatry, MHC Sct. Hans, Mental Health Services Copenhagen, Copenhagen, Denmark. [64]Institute of Clinical Sciences, Faculty of Medicine and Health Sciences, University of Copenhagen, Copenhagen, Denmark. [65]iPSYCH—The Lundbeck Foundation Initiative for Integrative Psychiatric Research, Copenhagen, Denmark. [66]http://www.headachegenetics.org/. [67]Department of Health, National Institute for Health and Welfare, Helsinki, Finland. [68]Research Centre of Applied and Preventive Cardiovascular Medicine, University of Turku, Turku, Finland. [69]Department of Clinical Physiology and Nuclear Medicine, Turku University Hospital, Turku, Finland. [70]Department of Neurology, Erasmus University Medical Center, Rotterdam, the Netherlands. [71]Department of Epidemiology and Biostatistics, MRC Health Protection Agency (HPE) Centre for Environment and Health, School of Public Health, Imperial College London, London, UK. [72]Biocenter Oulu, University of Oulu, Oulu, Finland. [73]Unit of Primary Care, Oulu University Hospital, Oulu, Finland. [74]Institute of Human Genetics, University Medical Center Hamburg-Eppendorf, Hamburg, Germany. [75]Population Health Research Institute, St George's, University of London, London, UK. [76]Munich Cluster for Systems Neurology (SyNergy), Munich, Germany. [77]Department of Human Genetics, Leiden University Medical Centre, Leiden, the Netherlands. [78]Faculty of Medicine, University of Iceland, Reykjavik, Iceland. [79]Statistical and Genomic Epidemiology Laboratory, Institute of Health and Biomedical Innovation, Queensland University of Technology, Kelvin Grove, Queensland, Australia. [80]Department of Neurology, Massachusetts General Hospital, Boston, Massachusetts, USA. [81]These authors contributed equally to this work. [82]These authors jointly supervised this work.

International Stroke Genetics Consortium (ISGC) Intracranial Aneurysm Working Group:

Masato Akiyama. Laboratory for Statistical and Translational Genetics, RIKEN Center for Integrative Medical Sciences, Yokohama, Japan; Department of Cancer Biology, Institute of Medical Science, The University of Tokyo, Tokyo, Japan. Department of Ophthalmology, Graduate School of Medical Sciences, Kyushu University, Fukuoka, Japan.
Varinder S. Alg. Stroke Research Centre, University College London, Institute of Neurology, London, UK.
Mark K. Bakker. Department of Neurology and Neurosurgery, University Medical Center Utrecht Brain Center, Utrecht University, Utrecht, The Netherlands.
Philippe Bijlenga. Neurosurgery Division, Department of Clinical Neurosciences, Faculty of Medicine, Geneva University Hospitals, Geneva, Switzerland
Sigrid Børte. K. G. Jebsen Center for Genetic Epidemiology, Department of Public Health and Nursing, Faculty of Medicine and Health Sciences, Norwegian University of Science and Technology, Trondheim, Norway; Research and Communication Unit for Musculoskeletal Health (FORMI), Department of Research, Innovation and Education, Division of Clinical Neuroscience, Oslo University Hospital, Oslo, Norway; Institute of Clinical Medicine, Faculty of Medicine, University of Oslo, Oslo, Norway.
Romain Bourcier. Université de Nantes, CHU Nantes, INSERM, CNRS, l'institut du thorax, Nantes, France; CHU Nantes, Department of Neuroradiology, Nantes, France.
Joseph P. Broderick. University of Cincinnati College of Medicine, Cincinnati, OH, USA.
Ben M. Brumpton. K. G. Jebsen Center for Genetic Epidemiology, Department of Public Health and Nursing, Faculty of Medicine and Health Sciences, Norwegian University of Science and Technology, Trondheim, Norway.
Zhengming Chen. Clinical Trial Service Unit and Epidemiological Studies Unit, Nuffield Department of Population Health, University of Oxford, Oxford, UK; Medical Research Council Population Health Research Unit, University of Oxford, Oxford, UK.
Jérôme Dauvillier. SIB Swiss Institute of Bioinformatics, Lausanne, Switzerland.
Hubert Desal. Université de Nantes, CHU Nantes, INSERM, CNRS, l'institut du thorax, Nantes, France.
CHU Nantes, Department of Neuroradiology, Nantes, France.
Christian Dina. Université de Nantes, CHU Nantes, INSERM, CNRS, l'institut du thorax, Nantes, France.
François Eugène. Department of Neuroradiology, University Hospital of Rennes, Rennes, France.
Mikael von Und Zu Fraunberg. Neurosurgery NeuroCenter, Kuopio University Hospital, Kuopio, Finland; Institute of Clinical Medicine, Faculty of Health Sciences, University of Eastern Finland, Kuopio, Finland.
Christoph M. Friedrich. Dortmund University of Applied Science and Arts, Dortmund, Germany.
Institute for Medical Informatics, Biometry and Epidemiology (IMIBE), University Hospital Essen, Essen, Germany.
Emília I. Gaál-Paavola. Department of Neurosurgery, Helsinki University Hospital, University of Helsinki, Helsinki, Finland and Clinical Neurosciences, University of Helsinki, Helsinki, Finland.
Jean-Christophe Gentric. Department of Neuroradiology, University Hospital of Brest, Brest, France.
Sven Hirsch. Zurich University of Applied Sciences, School of Life Sciences and Facility Management, Zurich, Switzerland.
Isabel C. Hostettler. Department of Neurosurgery, Kantonspital St. Gallen, Rorschacher Strasse 95, 9007, St. Gallen, Switzerland.
Henry Houlden. Neurogenetics Laboratory, The National Hospital of Neurology and Neurosurgery, London, UK.
Kristian Hveem. K. G. Jebsen Center for Genetic Epidemiology, Department of Public Health and Nursing, Faculty of Medicine and Health Sciences, Norwegian University of Science and Technology, Trondheim, Norway.

Juha E. Jääskeläinen. Neurosurgery NeuroCenter, Kuopio University Hospital, Kuopio, Finland. And Institute of Clinical Medicine, Faculty of Health Sciences, University of Eastern Finland, Kuopio, Finland.
Marianne Bakke Johnsen. K. G. Jebsen Center for Genetic Epidemiology, Department of Public Health and Nursing, Faculty of Medicine and Health Sciences, Norwegian University of Science and Technology, Trondheim, Norway; Research and Communication Unit for Musculoskeletal Health (FORMI), Department of Research, Innovation and Education, Division of Clinical Neuroscience, Oslo University Hospital, Oslo, Norway; Institute of Clinical Medicine, Faculty of Medicine, University of Oslo, Oslo, Norway.
Yoichiro Kamatani. Graduate School of Frontier Sciences, The University of Tokyo, Tokyo, Japan.
Masaru Koido. Laboratory for Statistical and Translational Genetics, RIKEN Center for Integrative Medical Sciences, Yokohama, Japan; Department of Cancer Biology, Institute of Medical Science, The University of Tokyo, Tokyo, Japan.
Liming Li. Department of Epidemiology, School of Public Health, Peking University Health Science Center, Beijing, China.
Kuang Lin. Clinical Trial Service Unit and Epidemiological Studies Unit, Nuffield Department of Population Health, University of Oxford, Oxford, UK.
Antti Lindgren. Neurosurgery NeuroCenter, Kuopio University Hospital, Kuopio, Finland.
Institute of Clinical Medicine, Faculty of Health Sciences, University of Eastern Finland, Kuopio, Finland.
Olivier Martin. SIB Swiss Institute of Bioinformatics, Lausanne, Switzerland.
Koichi Matsuda. Graduate School of Frontier Sciences, The University of Tokyo, Tokyo, Japan.
Laboratory of Clinical Genome Sequencing, Graduate School of Frontier Sciences, The University of Tokyo, Tokyo, Japan.
Iona Y. Millwood. Clinical Trial Service Unit and Epidemiological Studies Unit, Nuffield Department of Population Health, University of Oxford, Oxford, UK; Medical Research Council Population Health Research Unit, University of Oxford, Oxford, UK.
Sandrine Morel. Neurosurgery Division, Department of Clinical Neurosciences, Faculty of Medicine, Geneva University Hospitals, Geneva, Switzerland and Department of Pathology and Immunology, Faculty of Medicine, University of Geneva, Geneva, Switzerland.
Olivier Naggara. Pediatric Radiology, Necker Hospital for Sick Children, Université Paris Descartes, Paris, France; Department of Neuroradiology, Sainte-Anne Hospital and Université Paris Descartes, INSERM UMR S894, Paris, France.
Mika Niemelä. Department of Neurosurgery, Helsinki University Hospital, University of Helsinki, Helsinki, Finland.
Joanna Pera. Department of Neurology, Faculty of Medicine, Jagiellonian University Medical College, ul. Botaniczna 3, 31-503, Krakow, Poland.
Richard Redon. l'institut du thorax Université de Nantes, CHU Nantes, INSERM, CNRS, Nantes, France.
Gabriel J.E. Rinkel. Department of Neurology and Neurosurgery, University Medical Center Utrecht Brain Center, Utrecht University, Utrecht, The Netherlands.
Guy A. Rouleau. Montréal Neurological Institute and Hospital, McGill University, Montréal, QC, Canada.
Ynte M. Ruigrok. Department of Neurology and Neurosurgery, University Medical Center Utrecht Brain Center, Utrecht University, Utrecht, The Netherlands.
Marie Søfteland Sandvei. Department of Public Health and Nursing, Faculty of Medicine and Health Sciences, Norwegian University of Science and Technology, Trondheim, Norway; The Cancer Clinic, St Olavs Hospital, Trondheim University Hospital, Trondheim, Norway.
Sabine Schilling. Zurich University of Applied Sciences, School of Life Sciences and Facility Management, Zurich, Switzerland.
Eimad Shotar. Department of Neuroradiology, Pitié-Salpêtrière Hospital, Paris, France.
Agnieszka Slowik. Department of Neurology, Faculty of Medicine, Jagiellonian University Medical College, ul. Botaniczna 3, 31-503, Krakow, Poland.
Chikashi Terao. Department of Ophthalmology, Graduate School of Medical Sciences, Kyushu University, Fukuoka,
Jan H. Veldink. Department of Neurology and Neurosurgery, University Medical Center Utrecht Brain Center, Utrecht University, Utrecht, The Netherlands.
W. M. Monique Verschuren. Julius Center for Health Sciences and Primary Care, University Medical Center Utrecht, Utrecht, The Netherlands; National Institute for Public Health and the Environment, Bilthoven, The Netherlands.
Robin G. Walters. Clinical Trial Service Unit and Epidemiological Studies Unit, Nuffield Department of Population Health, University of Oxford, Oxford, U.K.; Medical Research Council Population Health Research Unit, University of Oxford, Oxford, U.K.
David J. Werring. Stroke Research Centre, University College London Queen Square Institute of Neurology, London, UK.
Cristen J. Willer. Department of Internal Medicine, Division of Cardiovascular Medicine, University of Michigan, Ann Arbor, MI, USA.
Bendik S. Winsvold. Department of Research, Innovation and Education, Division of Clinical Neuroscience, Oslo University Hospital, Oslo, Norway; K. G. Jebsen Center for Genetic Epidemiology, Department of Public Health and Nursing, Faculty of Medicine and Health Sciences, Norwegian University of Science and Technology, Trondheim, Norway.
Daniel Woo. University of Cincinnati College of Medicine, Cincinnati, OH, USA.
Bradford B. Worrall. Departments of Neurology and Public Health Sciences, University of Virginia School of Medicine, Charlottesville, VA, USA.
Sirui Zhou. Lady Davis Institute, Jewish General Hospital, McGill University, Montréal, QC, Canada.
John-Anker Zwart.
Department of Research, Innovation and Education, Division of Clinical Neuroscience, Oslo University Hospital, Oslo, Norway; K. G. Jebsen Center for Genetic Epidemiology, Department of Public Health and Nursing, Faculty of Medicine and Health Sciences, Norwegian University of Science and Technology, Trondheim, Norway; Institute of Clinical Medicine, Faculty of Medicine, University of Oslo, Oslo, Norway.
