# Supplementary material for: Genetic Risk Score for Intracranial Aneurysms: Prediction of Subarachnoid Hemorrhage and Role in Clinical Heterogeneity
Source: Stroke. 2023 Jan 19;54(3):810–8. doi: 10.1161/STROKEAHA.122.040715 (PMC9951795; doi:10.1161/STROKEAHA.122.040715)
Supplement: Supplementary file 3 [file str-54-810-s003.pdf]

Polygenic Risk Score Reporting Standard (PRS-RS)

Reporting standard		Description	Position in text
**Background**	**Study type**	We aimed to develop and validate a new polygenic risk score (PRS)	Figure 1, Methods paragraphs 'Methods overview', 'Constructing the metaGRS', and 'Prediction of ASAH and IA by the metaGRS' (page 7-10)
	**Risk model purpose and predicted outcome**	The PRS is intended to predict hazard of aneurysmal subarachnoid hemorrhage (ASAH), and presence of an intracranial aneurysm (IA). There are no existing prediction methods for these diseases. The PRS was also used to assess associations with, but not predict, clinical characteristics of IA.	Introduction paragraph 4 (page 6)
**Study population and data**	**Study design and recruitment**	UK Biobank: prospective cohort study. Nordic HUNT study: prospective cohort study. ISGC-IA phenotype cohort: case-only cohort study.	Methods paragraph 'Methods overview' (page 7)
	**Participant demographics and clinical characteristics**	See Table 1	-
	**Ancestry**	European ancestry individuals were included for constructing and for validation of the PRS	Methods paragraph 'constructing the metaGRS' (page 8-9)
	**Genetic data**	Genetic data was obtained by genotyping and imputation against the Haplotype Reference Consortium panel. More details in Bakker, et al. Nat Genet 2020;52(12):1303-13.	Methods paragraph 'Methods overview' (page 7)
	**Non-genetic variables**	No non-genetic variables were used in the model. These were only used to assess the added value of the PRS compared to non-genetic variables	-

Reporting standard		Description	Position in text
		sex, age, blood pressure, and smoking.	
	**Outcome of interest**	IA presence and age at ASAH. Selection based on ICD-10 codes, and exclusion criteria were connective tissue disorders, also selected by ICD-10 codes.	Methods paragraph 'Methods overview' (page 7), and Supplementary Data
	**Missing data**	Samples with a missing genotype were ignored for association of the specific variant, while samples with missing phenotype were excluded.	Methods paragraph 'Constructing the metaGRS' (page 8-9)
**Risk model development and application**	**PRS construction and estimation**	Optimal trait-level variant weights were obtained by comparing clumping (9 thresholds), sBayes R, and BLUP and picking the model with highest Nagelkerke pseudo R-squared for predicting IA. Trait-level weights were obtained by elastic net regression of the optimal trait-level model combined and optimizing the AUC for prediction of IA.	Methods paragraph 'Constructing the metaGRS' (page 8-9)
	**Risk model type**	Logistic regression for IA and ASAH presence. Cox regression for ASASH hazard. Risk reported in odds ratio and hazard ratio, respectively.	Methods paragraph 'Prediction of ASAH and IA by the metaGRS' (page 9-10) and Results paragraphs 'Prediction of ASAH by the metaGRS' (page 13-14) and 'Prediction of IA by the metaGRS' (page 14)
	**Integrated risk model(s) description and fitting**	Optimal trait-level variant weights for 17 traits genetically correlated with IA were obtained by comparing clumping (9 thresholds), sBayes R, and BLUP and picking the model with highest Nagelkerke pseudo R-squared for predicting IA. Trait-level weights were obtained by elastic net regression of the optimal trait-level model combined and optimizing the AUC for prediction of IA.	Methods paragraph 'Constructing the metaGRS' (page 8-9)
**Risk model evaluation**	**PRS distribution**	Min, max, mean and standard deviation are reported in Results paragraph 'Prediction of ASAH by the metaGRS'.	Page 13

Reporting standard		Description	Position in text
	**Risk model predictive ability**	Odds ratio and hazard ratio for prediction of IA presence and ASAH hazard for the metaGRS and non-genetic risk factors are given in Results paragraph 'Prediction of ASAH by the metaGRS' and 'Prediction of IA by the metaGRS'.	Results paragraph 'Prediction of ASAH by the metaGRS' (page 13-14) and 'Prediction of IA by the metaGRS' (page 14)
	**Risk model discrimination**	C-index and AUROC are given in 'Prediction of ASAH by the metaGRS' and 'Prediction of IA by the metaGRS'.	Results paragraph 'Prediction of ASAH by the metaGRS' (page 13-14) and 'Prediction of IA by the metaGRS' (page 14)
	**Risk model calibration**	No calibration was performed	-
	**Subgroup analyses**	Subgroup size for IA and ASAH are given in Table 1. Subgroup sizes for the associations between metaGRS and clinical characteristics are given in Table 2.	-
**Limitations and clinical implications**	**Risk model interpretation**	A summary of what the risk model predicts and how well, the performance of the metaGRS alone, and its relation to non-genetic risk factors are discussed in Discussion paragraph 1.	Discussion paragraph 1 (page 17)
	**Limitations**	Limitations of the study include limited prediction of IA due to many undetected IA in the population (Discussion paragraph 2), overlap with variance captured by non-genetic risk factors (Discussion paragraph 5), and differences in genetic risk between men and women (Discussion paragraph 6).	Discussion paragraph 2 (page 17), paragraph 5 (page 19), and paragraph 6 (page 19)
	**Generalizability**	Generalizability to sexes and rupture status are discussed in Discussion paragraphs 2 and 6.	Discussion paragraph 5 (page 19) and paragraph 6 (page 19)
	**Risk model intended uses**	Predictive performance currently is insufficient to be used for clinical prediction. This is stated in Discussion paragraphs 1 and 7.	Discussion paragraph 1 (page 17) and paragraph 7 (page 19)

Reporting standard	Description	Position in text
**Data transparency and availability**	Variant-level scores for the metaGRS are publicly available without restriction here: https://doi.org/10.6084/m9.figshare.19672272 (including UK Biobank) and here: https://doi.org/10.6084/m9.figshare.19672269 (excluding UK Biobank).	-
